# Seasonal transition in snow effects on vegetation growth across the Tibetan Plateau

**DOI:** 10.3389/fpls.2026.1855382

**Published:** 2026-07-13

**Authors:** Zekai Meng, Xiuchen Wu, Yongmei Huang, Renjie Guo, Xiaona Wang, Wenqi Song, Zifan Zhang

**Affiliations:** 1 State Key Laboratory of Earth Surface Processes and Disaster Risk Reduction, Beijing Normal University, Beijing, China; 2 Faculty of Geographical Science, Beijing Normal University, Beijing, China; 3 Department of Earth System Science, Ministry of Education Key Laboratory for Earth System Modeling, Institute for Global Change Studies, Tsinghua University, Beijing, China; 4 Department of Health and Environmental Sciences, School of Science, Xi’an Jiaotong-Liverpool University, Suzhou, China

**Keywords:** alpine ecosystem, phenology, snow water equivalent, snowmelt date, soil moisture, vegetation growth

## Abstract

Snow exerts intricate influences on alpine ecosystems, and winter snow process is undergoing drastic change with climate warming. Yet the impacts of winter snow on vegetation growth (hereafter, snow effects) and their underlying mechanisms remain uncertain, as snow effects operate through multiple pathways. Based on remote sensing-derived snow and vegetation products, we examine the snow effects on vegetation growth in spring and summer across the Tibetan Plateau through partial correlation analysis. We then quantify the distinct pathways through which snow influences vegetation growth in spring and summer using pixel-wised piecewise-Structural Equation Modeling, and further identify the transitions of dominant ecological processes underlying these effects. We report a marked seasonal transition of snow effects on vegetation growth, with negative-to-positive shifts observed in 25% of the Tibetan Plateau from spring to summer. The negative snow effects in spring are mainly attributable to the snowmelt-date (SMD)–induced phenological pathway, through which earlier snowmelt advances spring phenology and thereby influences vegetation growth. In contrast, snow effects in summer are predominantly positive due to the reversal of snow-induced phenological effects from spring and the carryover of snow-driven soil moisture effects into summer, which promotes vegetation growth. Finally, we found that most of the dynamic global vegetation models (DGVMs) used in this study have limited ability to reproduce this seasonal transition in snow effects. These findings highlight the critical seasonal shifts in ecological processes that underpin snow effects on ecosystems, providing valuable insights into improving ecosystem models.

## 1 Introduction

Hydrological cycle on the Tibetan Plateau (TP), known as the “Asian water tower”, is being profoundly perturbed by drastic climate change. Seasonal snow storage and the timing of snowmelt are highly sensitive to warming, increasing the likelihood of snow droughts and threatening water security for both ecosystems and human use, particularly in snow-dominated alpine grasslands. Dramatic changes in snow cover phenology, including onset, end date, and duration, have been observed by both remote sensing (Li et al., 2022; Yang et al., 2019) and *in situ* observations (Xu et al., 2024; Zhang et al., 2013). These shifts in snow dynamics are closely aligned with decades-long warming trends and localized changes in precipitation (Xu et al., 2024). Since 2008, a critical slowdown in snow cover has been reported on the TP, with some regions approaching a ‘zero snow dominated duration’ (Allchin and Déry, 2017), suggesting that winter snow processes are likely approaching a tipping point (Liu et al., 2023b). Moreover, the TP has been identified as one of the global hotspots for snow drought in recent decades (Huning and AghaKouchak, 2020). Yet snow cover remains critical for sustaining agricultural water supply and vegetation growth across the TP and adjacent regions, both of which are under mounting pressure (Barnett et al., 2005; Mankin et al., 2015; Qin et al., 2020).

Pre-growing season conditions are critical for shaping alpine ecosystem processes, particularly in snow-dominated regions where snow controls both energy and water fluxes (Van Wijk and Williams, 2003; Wu et al., 2022). Some studies even suggest that winter conditions may be more influential than those in spring or summer in determining ecosystem fluxes (Bjerke et al., 2015; Brooks et al., 2011), largely due to the multifaceted effects of snow on vegetation growth. Physically, the insulating properties of snow maintain relatively warm soil temperatures, preventing root damage from freezing (Grippa et al., 2005; Reinmann et al., 2019). Snowpack also exerts strong hydrological and albedo effects, triggering climate feedbacks that extend into the subsequent growing season (Bamzai and Shukla, 1999; Su et al., 2013; Wipf et al., 2009; Xu and Dirmeyer, 2011; You et al., 2020). Biologically, earlier snowmelt can advance spring greening, flowering, and reproduction (Borner et al., 2008; Cooper et al., 2011; Frei and Henry, 2022; Natali et al., 2012; Richardson et al., 2013), thereby extending the growing season and increasing productivity (Borner et al., 2008; Wang et al., 2018). However, early phenology induced by advanced snowmelt may expose plants to accidental cold air temperatures, resulting in frost damage or inhibited growth (Wipf et al., 2006; Høye et al., 2007; Inouye, 2008; Kelsey et al., 2021). The “chilling requirement” hypothesis further suggests that advanced snowmelt can delay bud break in spring (Pua and Davey, 2010; Schwartz, 2013). Limited evidence also indicates that while snowmelt timing determines the onset of phenological phases, the duration of the growing season remains relatively fixed (Semenchuk et al., 2016). Beyond phenological effects, winter snow enhances water availability in the growing season, directly supplying soil water for vegetation growth (Brooks et al., 2011; Grippa et al., 2005; Moriana-Armendariz et al., 2022; Rumpf et al., 2014), thereby leading to a positive correlation between snow depth and vegetation greenness (Grippa et al., 2005; Wang et al., 2018). In addition, snow cover and snowmelt timing regulate thaw depth and soil temperature in winter, indirectly influencing microbial communities that control nutrient cycling, thereby affecting vegetation growth in subsequent seasons (Borner et al., 2008; Brooks et al., 2011). Snow effects can also persist beyond the timing of snowmelt through biological and hydrological carry-over effects, whereby conditions established in earlier seasons influence vegetation growth in subsequent periods. Such carry-over effects, particularly via soil moisture and plant physiological states, may extend the influence of snow from spring into summer. Despite these well-documented pathways, there is still no consensus on how vegetation growth responds to winter snow changes, as snow influences vegetation growth through multiple, sometimes competing pathways, resulting in complex and often inconsistent ecological outcomes. We therefore hypothesize that snow regulates vegetation growth through seasonally shifting ecological pathways, leading to contrasting effects between spring and summer. In spring, greater snow accumulation is expected to delay snowmelt and consequently postpone phenological development, thereby constraining vegetation growth and resulting in negative effects. In contrast, in summer, positive effects are expected to emerge from the carry-over of snow-derived soil moisture and this phenology shifts.

Several challenges continue to hinder our understanding of the snow–productivity relationships. One key challenge lies in disentangling snow effects into individual pathways. Much of the previous research on snow–vegetation interactions has focused on lagged effects of winter snow. Approaches based solely on maximum correlations introduce large uncertainties, as snow exerts multifaceted and often opposing influences on vegetation growth. For example, in summer, both snow depth and snowmelt date exhibit positive relationships with vegetation growth. However, snowmelt date is negatively correlated with vegetation growth over the growing season, whereas snow depth remains positively correlated (Grippa et al., 2005; Wang et al., 2018). This pattern indicates a seasonal shift in snow–vegetation relationships. In contrast, snow depth-induced effects dominate in summer due to soil moisture memory or carryover effects of vegetation (Lian et al., 2021). In addition, climatic factors such as precipitation and temperature at different developmental stages interact with snow cover, further complicating the pathways through which snow influences vegetation growth (Høye et al., 2007; Jonas et al., 2008; Kelsey et al., 2021; Frei and Henry, 2022). Vegetation growth responses to snow dynamics also vary widely across ecosystem types (Park et al., 2019). Consequently, despite substantial progress, a comprehensive understanding of the seasonal transitions and the dominant pathways through which snow affects vegetation growth is still lacking.

Here, we disentangled the complex snow effects and quantified their seasonal transitions on the Tibetan Plateau by combining remotely sensed Normalized Difference Vegetation Index (NDVI) with gridded snow reanalysis products and climate data. We further investigated and compared the snow effects in six global ecosystem models in order to comprehensively evaluate the parameterizations of winter snow processes in these state-of-the-art models. Partial correlation analysis was applied to isolate snow effects from other climatic drivers. To further clarify the underlying pathways, we developed piecewise Structural Equation Models (SEMs) for spring and summer, thereby disentangling the ecological pathways through which winter snow influences vegetation growth. This framework enabled us to reveal the temporal and spatial patterns in snow effects via diverse pathways and to attribute seasonal transitions to shifts in dominant processes. Specifically, we addressed two questions: (1) Does snow effect on vegetation growth exhibit seasonal transitions? (2) If so, which ecological processes drive these transitions?

## 2 Materials and methods

### 2.1 Study area

This study was conducted on the Tibetan Plateau (TP), which covers a total area of approximately 3.0834 million km^2^, with a mean elevation of about 4,320 m. Influenced by high elevation, temperatures are generally low, with small annual variability but large diurnal ranges, and mean annual temperature ranges from −33 to 21 °C. Warmer conditions occur south of the Great Bend of the Yarlung Zangbo River and in the Three-River Source Region, whereas the plateau interior is colder. Precipitation shows strong seasonality, with a wet season from April to September and a dry season from October to March, and exhibits substantial spatial heterogeneity. Seasonal snow covers most of the TP. Regions with heavy snowfall, mainly located in the mountainous areas of the southern and southwestern TP and the Pamir Plateau, are characterized by high snow water equivalent (**Supplementary Figure 1b**). It hosts diverse vegetation types and high species diversity. From east to west, the dominant vegetation types include temperate coniferous forests, alpine shrublands, alpine meadows, and alpine steppes (**Supplementary Figure 1a**). Alpine meadows and steppes are the predominant vegetation types, accounting for approximately 55% of the total area. Forests are mainly distributed in the Hengduan Mountains and the Himalayas, while shrublands occur in the transitional zones surrounding forests and in the northeastern part of the Plateau.

### 2.2 Observational datasets

Daily snow water equivalent (SWE) data at 500 m spatial resolution were obtained from the High Mountain Asia Snow Reanalysis (HMASR) database for the period 2000–2017 (Liu et al., 2021; Lalande et al., 2023) (https://nsidc.org/data/hma_sr_d/versions/1). This dataset contains gridded data organized by water year (WY, e.g., WY 2001 spanning from 1 October 2000–30 September 2001). As part of the NASA High Mountain Asia Team (HiMAT) initiative, the HMASR was developed using a Bayesian snow reanalysis scheme, with input from Landsat and MODIS images. The dataset is more accurate than other reanalysis datasets and has been used as a reference for snow water equivalent research on the Tibetan Plateau (Liu et al., 2022). To investigate regional variations in snow storage and the impact of snowpack water availability, analyses were restricted to areas with seasonal snow, excluding permanent snow, ice sheets, and ephemeral snow due to their intermittent accumulation and ablation within a water year. The pixel-wise peak SWE (SWE_peak_) was used as a proxy for maximum water availability from snowpacks in each water year. SWE_peak_ represents the transition between accumulation and ablation phases, capturing winter’s maximum water content (Liu et al., 2022). We resampled SWE_peak_ to 0.05° using average method to match the spatial resolution of vegetation index. To delineate snow ablation timing, the pixel-wise snowmelt date (SMD) was defined for each water year as the final day of the last five consecutive days during which daily SWE remained above 1 mm (Peng et al., 2010; Wang et al., 2018).

Vegetation growth was characterized using the Normalized Difference Vegetation Index (NDVI) derived from the MODIS Collection 6 MOD13A2 product, provided every 16 days at 1 km resolution for the period 2000 – 2017 (Didan, 2015, https://lpdaac.usgs.gov/products/mod13a2v006/). MOD13A2 includes QA layers and Day-of-Year (DOY) information at the pixel level, facilitating precise phenological analyses and the removal of cloud- or snow-contaminated pixels. NDVI and QA layers were resampled to a spatial resolution of 0.05° using averaging for NDVI and nearest-neighbor resampling for QA. Grid cells with multi-year (2000 - 2017) average NDVI < 0.1 were excluded from the final analyses to minimize the influence of sparsely vegetated areas. NDVI was used both for phenological extraction and quantification of vegetation growth. In addition to NDVI, we also used clear-sky daily solar-induced fluorescence (SIF) from the contiguous SIF (CSIF) dataset, available at 0.05° spatial resolution and four-day temporal resolution for 2000–2017 (https://data.tpdc.ac.cn/zh-hans/data/d7cccf31-9bb5-4356-88a7-38c5458f052b/), providing a more direct proxy of vegetation productivity closely correlated with gross primary production (GPP).

Climatic drivers potentially influencing vegetation growth were obtained from TerraClimate (Abatzoglou et al., 2018; https://www.climatologylab.org/terraclimate.html), including monthly temperature, shortwave radiation, vapor pressure deficit (VPD), and precipitation at 4 km resolution in the period of 2000–2017. These variables allow the separation of snow effects from other climatic influences (see Section 2.4, 2.5 for details), which are known to strongly affect vegetation growth on the Tibetan Plateau (Ding et al., 2018; Wang et al., 2023). Soil moisture (SM) was included as a direct indicator of soil water availability, using root-zone (0–10 cm, 10–40 cm) monthly data from FLDAS v4 (Noah 3.6.1 model; McNally et al., 2017) at 0.1° spatial resolution (https://disc.gsfc.nasa.gov/datasets/FLDAS_NOAH01_C_GL_M_001/summary). These shallow soil layers correspond to the rooting depths of dominant TP vegetation, including graminoids, forbs, and shrubs (Dorji et al., 2013; Hu et al., 2013; Miehe et al., 2019). All climatic factors were resampled to 0.05° using nearest-neighbor method to match others.

Vegetation type data was obtained from the 1:1,000,000-scale Vegetation Atlas of China (1980s) (Zhang, 2007; Zhou et al, 2022, https://data.tpdc.ac.cn/zh-hans/data/0b32907c-9f64-407b-8099-849db1900005). Major vegetation types include alpine meadow, alpine steppe, shrubland, broad-leaved forest, coniferous forest, mixed coniferous–broad-leaved forest, and alpine sparse vegetation. Forest categories (broad-leaved, coniferous, mixed) were combined into a single “forest” class. The vector vegetation data were resampled to a 0.05° grid using the maximum proportion method to align with the spatial resolution of other datasets.

### 2.3 Determination of phenophases and season division

In order to elucidate the potential mechanisms behind the temporal transition of snow impacts on vegetation growth, two seasons (hereafter, spring and summer) within the growing season are defined using NDVI series. Given the pronounced spatial heterogeneity of climate across the Tibetan Plateau (ranging from xeric steppe to moist forests), a spatially dynamic seasonal division was adopted. Nevertheless, the seasonal transitions in snow effects remain robust when using a fixed definition of spring and summer (i.e., April–June for spring and July–August for summer). For each location, we defined period of spring and summer based on its NDVI trajectory. First, a rough smoothing of the NDVI series was applied using a weighted fitting approach that incorporates NDVI quality assurance (QA) layers to minimize contamination from clouds and snow (Eilers, 2003; Frasso and Eilers, 2015; **Supplementary Figure 2**). QA weights were assigned as follows: good = 1, marginal = 0.5, snow/cloud = 0.2. Next, four advanced smoothing methods were applied to the NDVI series to enhance phenological signal extraction: asymmetric Gaussian, piecewise logistic (Zhang et al., 2003), double logistic (Beck et al., 2006), and nonlinear inverse modeling (Elmore et al., 2012). We then averaged the NDVI series across the four smoothing methods. The smoothed NDVI series were then used to extract pixel-wise phenological metrics, including the upturn day (UD), start of the growing season (SOS), and end of the growing season (EOS) (Gu et al., 2009). The UD is defined as the intersection between the recovery line and the x-axis, where the recovery line passes through the point of maximum early-season growth with a slope corresponding to the peak recovery rate (Gu et al., 2009). The SOS and EOS correspond to the dates when NDVI reaches 50% of its seasonal maximum in the early and late growing seasons, respectively. Grid cells exhibiting multiple growing seasons in a single year or lacking clear phenological cycles (e.g., evergreen forests) were excluded. Multi-year averages of UD, SOS, and EOS were then calculated and converted to monthly metrics (UD_m_, SOS_m_ and EOS_m_) to match the monthly resolution of climate variables. Spring was defined as the period of [UD_m_, SOS_m_], and summer as (SOS_m_, EOS_m_). For instance, for a pixel with UD_m_ = 4, SOS_m_ = 5 and EOS_m_ = 8, spring corresponds to April–May and summer to June–July (**Supplementary Figure 3**). Notably, spring was defined from UD rather than the commonly used SOS to capture the pre-flush period, which is critical for linking pre-growing-season snowpack and climate in subsequent modeling. The trends of SOS and EOS are both in agreement with previous findings on phenology in the Tibetan Plateau (**Supplementary Figure 4**). The whole processes were performed using the R package *phenofit* (Kong et al., 2022).

### 2.4 Quantifying snow effects on vegetation growth

Before examining the seasonal shifts in snow effects on vegetation growth, we first conducted a lagged partial correlation analysis between annual SWE_peak_ and subsequent NDVI. Specifically, the pixel-wise SWE_peak_ and its timing (SWE_peak_ DOWY, day of water year) were extracted for each water year. NDVI values were then obtained for 1–5 months following the SWE_peak_ DOWY using linear interpolation of the smoothed NDVI series. To account for the influence of other climatic factors, monthly temperature, insolation, vapor pressure deficit (VPD), and precipitation were similarly interpolated to match these lagged dates and included as covariates to isolate the effect of SWE_peak_.

To further assess snow impacts across seasons, partial correlations were performed between annual SWE_peak_ and seasonal mean NDVI in spring and summer. For each pixel, all vegetation growth and climatic variables were first aggregated into seasonal metrics: seasonal SIF, temperature, shortwave radiation, and VPD were calculated as seasonal averages, while seasonal precipitation was summed. Seasonal NDVI was computed as the mean NDVI within each season. In spring, partial correlations between SWE_peak_ and NDVI were computed while conditioning on spring temperature, precipitation, VPD, and shortwave radiation. In summer, partial correlations were similarly calculated while conditioning on summer climatic variables, with spring precipitation additionally included to account for lagged effects. We further performed partial correlation analysis between SWE_peak_ and NDVI over the entire growing season to investigate overall effects of snow. Growing-season NDVI was calculated as the mean NDVI from May to October, which covers most pixels across the Tibetan Plateau (**Supplementary Figure 3**).

### 2.5 Disentangling the seasonal shifts in snow effects on vegetation growth

We employed piecewise structural equation modeling (R package piecewiseSEM; Lefcheck, 2015) to disentangle the relationships between SWE_peak_ and vegetation growth across the TP, while simultaneously accounting for the effects of diverse climatic variables, including soil moisture, temperature, precipitation, and shortwave radiation. Piecewise SEM is more flexible with respect to sample size requirements and can accommodate complex ecological data structures. Pixel-wise SEMs were constructed separately for spring and summer, assuming linear relationships among variables. For each pixel, climatic variables were first aggregated into seasonal metrics following the same procedure as in the partial correlation analysis, and then incorporated into the SEM. The effect of SWE_peak_ on vegetation growth (hereafter, snow effect) was evaluated through a framework that considers phenological effects, soil moisture effects, and vegetation carry-over effects, all of which are supported by previous studies (Liu et al., 2023a; Wang et al., 2018). Potential interactions between SWE_peak_ and other climatic variables (temperature, precipitation, VPD and shortwave radiation) were also included. In piecewise SEM, pathways are not independent. This means that, on one hand, the influence of temperature on spring phenology—which is coupled with snow—is largely accounted for; on the other hand, including these climatic covariates allows the snow effect to be isolated, so that each indirect effect represents the net impact of SWE_peak_ on NDVI through that specific pathway, while other pathways are controlled.

To ensure robust estimation, SEMs were only constructed for pixels with at least 15 valid annual observations. Two prototype SEMs were initially built for spring and summer to capture the main ecological processes across the TP. These models were then applied to all pixels and iteratively refined. Specifically, candidate direct pathways that were not initially included were tested across all pixels, and a pathway was incorporated into the model if it met the following criteria: (1) it was statistically significant (p < 0.05) in more than 30% of pixels, and (2) its direction and interpretation were ecologically consistent with established knowledge. This procedure was repeated until no additional pathways satisfied these criteria. Spring and summer models were tuned independently. Direct pathways were manually determined, while indirect pathways were automatically derived once the model structure was established. Results derived from solar-induced fluorescence (SIF) was compared to further validate the results of NDVI.

Direct and indirect effects of each pathway were quantified. Direct effects correspond to the standardized coefficients from the SEM summary. Indirect effects of SWE_peak_ on NDVI —through phenology, soil moisture, and carry-over pathways—were calculated by multiplying the relevant direct effects along each causal path. Only indirect effects originating from climatic variables (e.g., SWE_peak_, seasonal temperature) and terminating at vegetation growth were considered. Tracing all indirect effects was performed using the *igraph* package in R.

Dominance analysis was conducted for each season. For each pixel, the pathway of SWE_peak_ with the largest absolute effect on vegetation growth was considered the dominant effect. Based on dominant effects in spring and summer, pixels were classified into three transition types: positive transition (dominant effect of snow effects changes from negative to positive from spring to summer), negative transition (dominant effect changes from positive to negative), and stable (direction of dominant effect remains unchanged from spring to summer). Model performance was evaluated using Fisher’s C-test (significant when 0.05 < *p* < 1.0) and the goodness-of-fit (R^2^).

### 2.6 TRENDY model simulations

We further investigated and compared the winter snow effects on vegetation productivity using simulations of global ecosystem models. We used simulations from six state-of-the-art ecosystem models involved in the TRENDY v.12 project, including CABLE-POP, CLASSIC, JULES, LPJmL, LPX-Bern, and SDGVM (Friedlingstein et al., 2024; Sitch et al., 2024, https://mdosullivan.github.io/GCB/). All models provide prognostic estimates of monthly GPP, climatic variables (temperature, downward shortwave radiation, precipitation) and snow-related variables (only one model provided snow water equivalent, while others provided snow depth). Data from each model were obtained under the S3 scenario, which considered all historical forcings (climate change, CO_2_ and land use change). All TRENDY model outputs were first resampled to 0.05° using nearest-neighbor method to match observational results. Monthly snow depth was used to derive annual peak snow depth (snow depth_peak_) for each water year (WY), defined as the maximum monthly snow depth within each WY. Monthly climatic variables and GPP were aggregated to seasonal value following the same season definitions as applied for NDVI analyses. Partial correlations between snow depth_peak_ and seasonal mean GPP were then calculated following the same protocol as the remote-sensing analyses. For models that provided snow depth separately for different plant functional types (PFTs) (i.e., CABLE-POP and SDGVM), partial correlations were first calculated at the PFT level and subsequently aggregated to the grid-cell scale using area-weighted averaging based on the fractional coverage of each PFT within a pixel.

## 3 Results

### 3.1 Seasonal transition of snow effects

**Figure 1:**
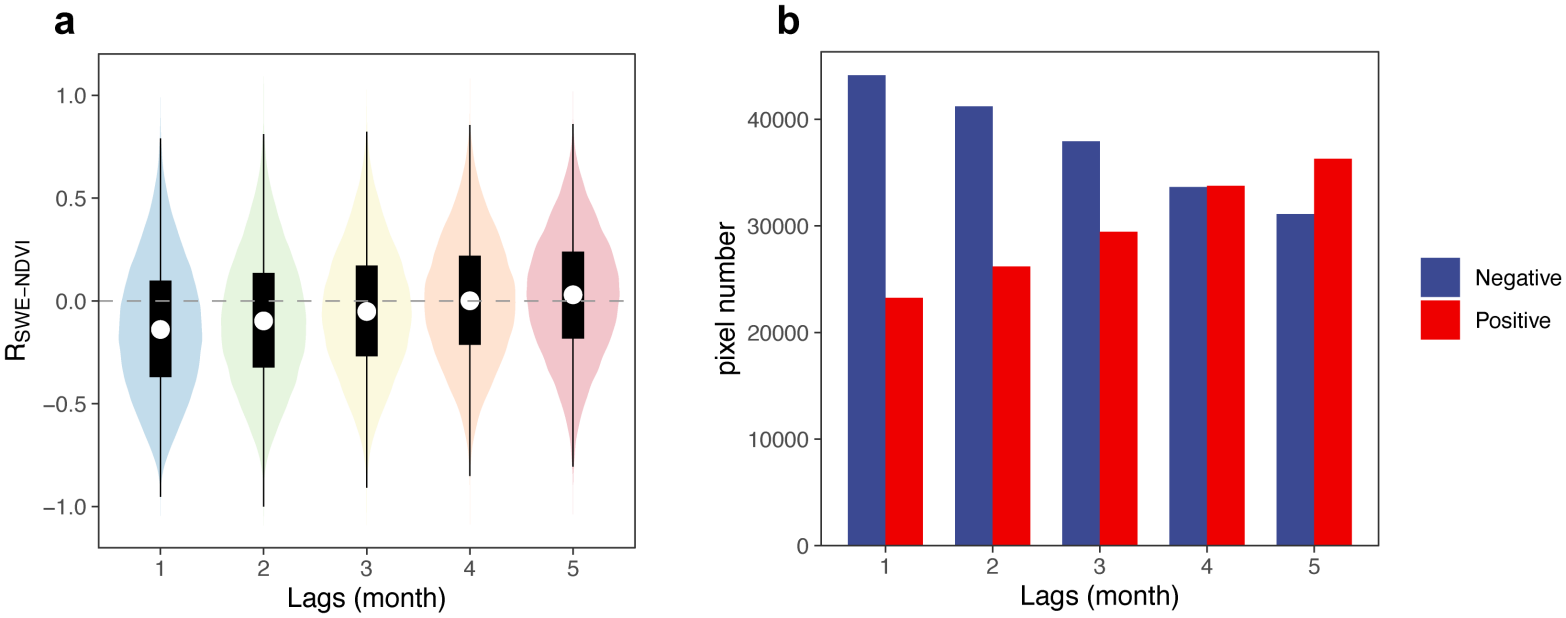
Lagged effects of SWE_peak_ on vegetation growth. **(a)** Partial correlation coefficient (R_SWE-NDVI_) under different lagged months over the TP. **(b)** Absolute number of samples showing positive or negative correlations at different lags. Negative, negative correlation; Positive, positive correlation.

We first assessed the lagged partial correlations between SWE_peak_ and monthly NDVI (lags of 1–5 months after SWE_peak_ DOY) across the TP during 2000–2017. The spatially averaged SWE_peak_–NDVI correlation coefficients increased with longer lags (y = 0.04x – 0.17, R^2^ = 0.98; **Figure 1a**). The number of pixels with positive correlations rose with lag time, while negatively correlated pixels declined (**Figure 1b**).

**Figure 2:**
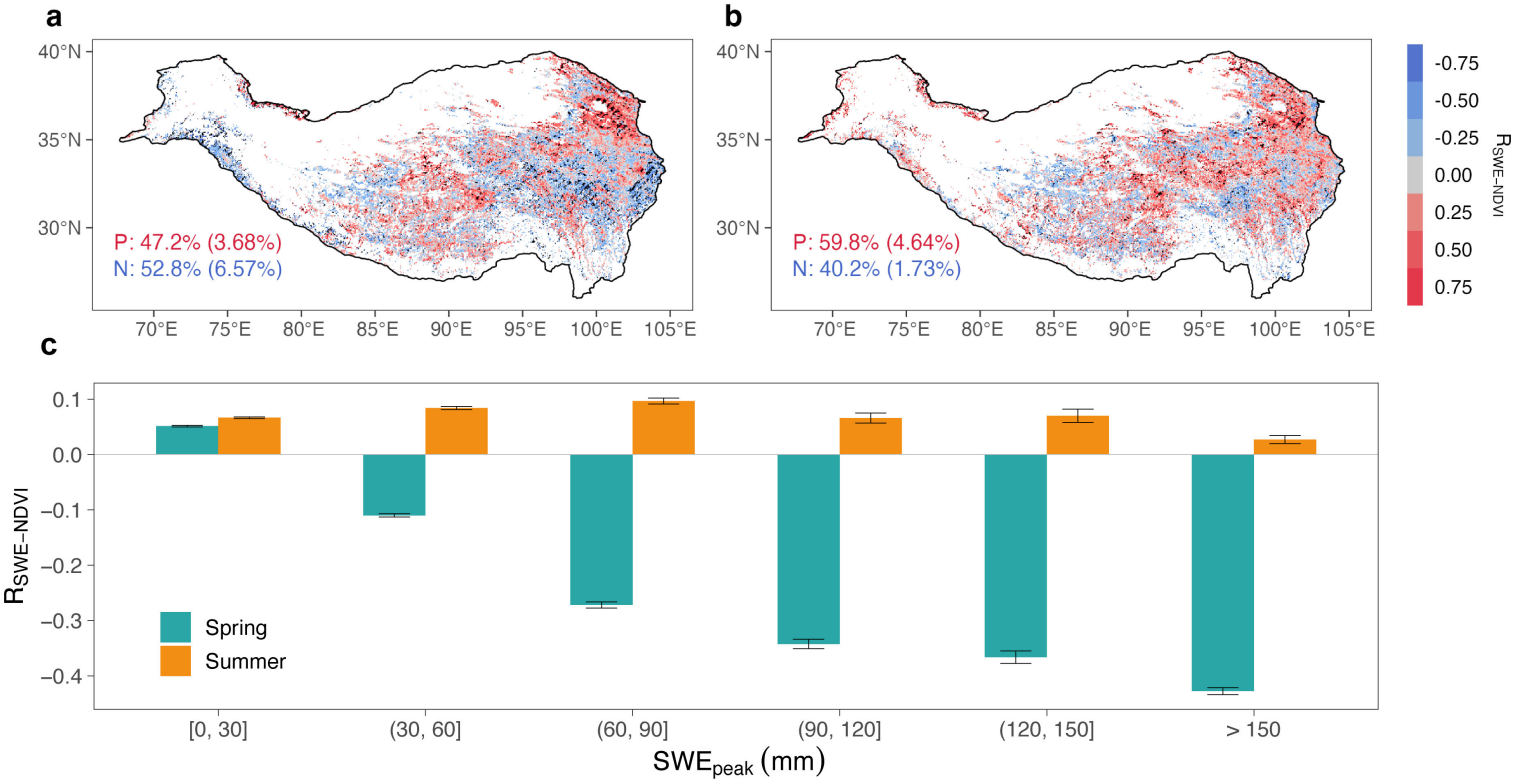
Seasonal differences in the effects of snow on NDVI. Spatial patterns of the partial correlation coefficient between SWE_peak_ and NDVI (R_SWE-NDVI_) in spring **(a)**, summer **(b)**, and its variation along the climatological SWE_peak_ gradient **(c)** (left to right, n = 35,561, 10,367, 3091, 1181, 587, 1855 for both spring and summer). The spring analysis controls for concurrent temperature, VPD, precipitation, and shortwave radiation, while the summer analysis additionally accounts for precipitation in the previous season. P, positive correlation; N, negative correlation. In each panel, overall percentages of positive/negative correlations are given, with significant proportions in parentheses. Areas with line patterns indicate significant R_SWE–NDVI_ relationships (P < 0.05). Error bars show 1 s.e.

A clear seasonal shift in snow effects on vegetation growth was observed from spring to summer. In spring, SWE_peak_ was negatively correlated with seasonal mean NDVI across 52.8% of the Tibetan Plateau (6.57% significant), whereas in summer, positive correlations predominated (59.8%, 4.64% significant; **Figure 2**). In addition, the strength of these relationships increased with climatological SWE, with more pronounced negative effects in spring and stronger positive effects in summer (**Figure 2c**). Significantly negative correlations in spring were located in parts of the eastern TP, central TP, and western Himalayas (**Figure 2a**), whereas significantly positive correlations in summer appeared in the southern Qinghai Lake basin, western Kunlun Mountains, and Pamir Plateau (**Figure 2b**). The snow effects on growing season NDVI further corroborate these seasonal patterns, with spatial signals persisting across seasons: regions exhibiting significantly negative correlations in spring tend to remain negatively correlated over the entire growing season, whereas areas with significantly positive correlations in summer also show positive relationships at the growing season scale (**Supplementary Figure 6**). Overall, 25% of the TP showed a negative-to-positive transition from spring to summer, while 12.4% showed a positive-to-negative shift. SIF-based results corroborated these patterns, consistently capturing a strong spring-to-summer transition (**Supplementary Figure 7**).

### 3.2 Seasonal role of snow effects associated with distinct ecological processes

We next disentangled the pathways mediating snow effects on vegetation growth using piecewise structural equation models (SEMs) for spring and summer, respectively (**Supplementary Figure 8**). The structure of SEMs are statistically robust for 92% of pixels in spring and 86.8% in summer (Fisher’s C-test *p* > 0.05) and explained a large fraction of NDVI variability (spring R^2^ = 0.65 ± 0.16; summer R^2^ = 0.76 ± 0.16).

**Figure 3:**
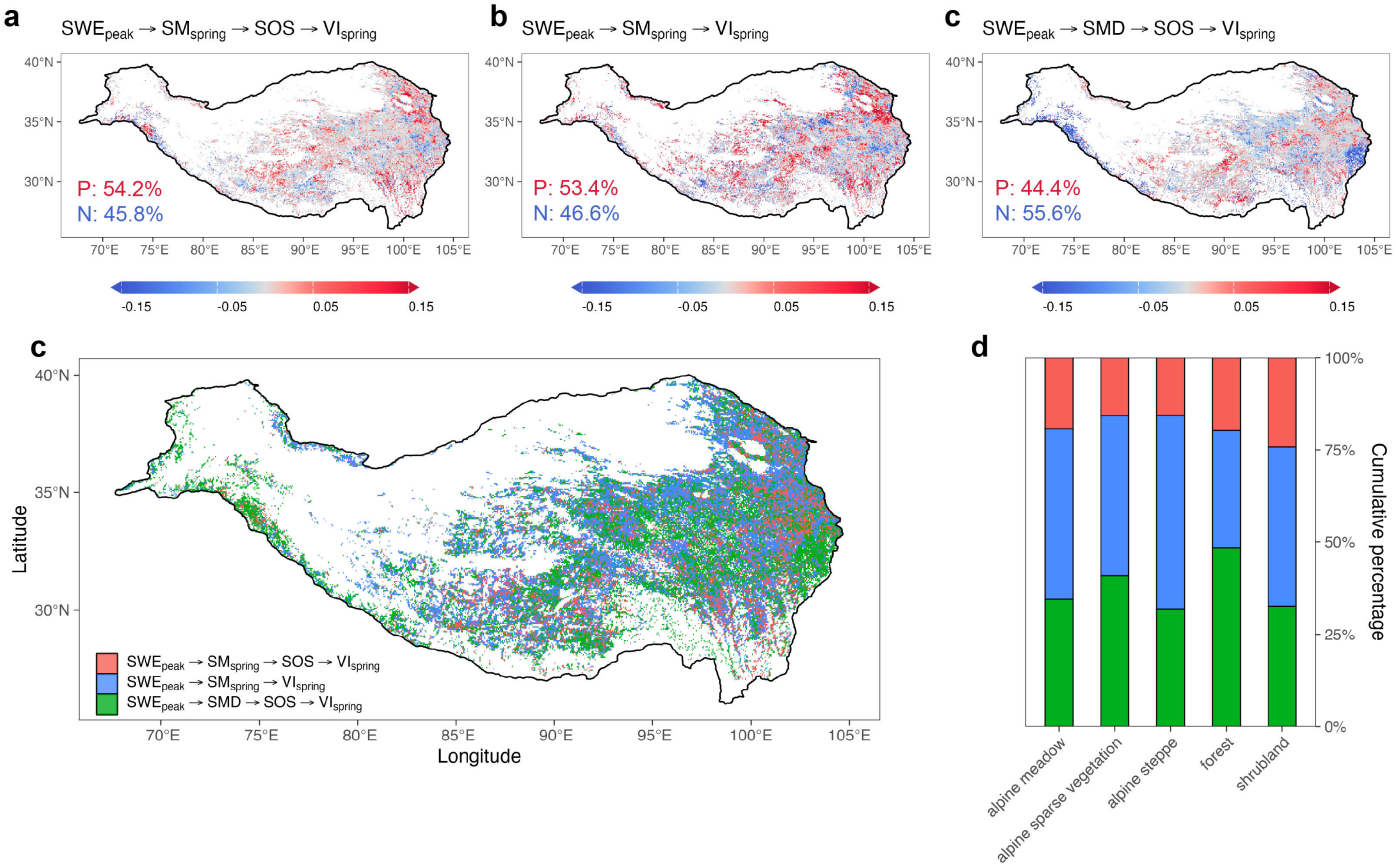
Pathways through which snowmelt influences NDVI in spring. Spatial patterns of indirect effects of SWE_peak_ on NDVI via different pathways **(a–c)**, calculated by multiplying the relevant direct effects along each causal path), spatial pattern of dominant snow effect types **(d)**, and their percentage across vegetation types **(e)**. P, positive effect; N, negative effect. SOS denotes the start of the growing season, VI denotes NDVI, SMD indicates the snowmelt date, and SM represents soil moisture.

**Figure 4:**
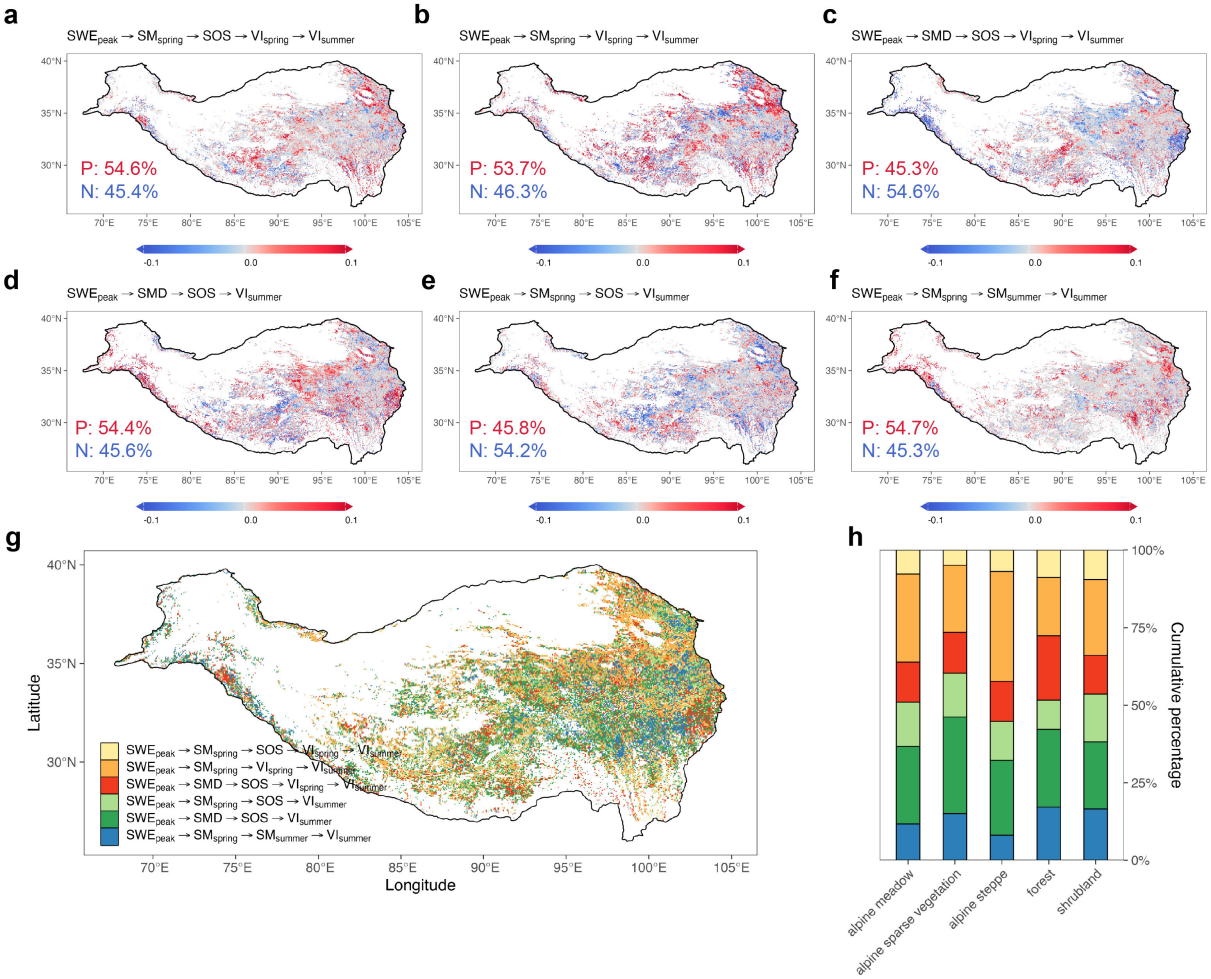
Pathways through which snowmelt influences NDVI in summer. Spatial patterns of indirect effects of SWE_peak_ on NDVI via different pathways **(a–f)**, calculated by multiplying the relevant direct effects along each causal path), spatial pattern of dominant snow effect types **(g)**; warm colors indicate carry-over effects of spring snow), and their percentage across vegetation types **(h)**. P, positive effect; N, negative effect. SOS denotes the start of the growing season, VI denotes NDVI, SMD indicates the snowmelt date, and SM represents soil moisture.

In spring, snow effects operated through three main pathways: (i) SMD-induced phenological pathway: SWE_peak_ delayed snowmelt and thereby spring phenology, resulting in negative path coefficients for NDVI. This constraint effect was most pronounced in the eastern TP and western Himalayas (**Figure 3c**). (ii) Soil moisture pathway: SWE_peak_ increased spring soil moisture, which promoted vegetation growth. Positive path coefficients were concentrated in the northeastern Qinghai Lake basin and the central TP (**Figure 3b**). (iii) Soil moisture (SM)-induced phenological pathway: SWE_peak_ altered soil moisture, which in turn affected spring phenology and hence NDVI. This pathway is dominated with positive path coefficients but had relatively limited dominance across TP (18.9%) (**Figure 3a**). At each pixel, we identified the dominant pathway as the one with the largest absolute effect (**Figures 3d**, **4g** and **S4**). The spatial patterns of dominant pathways derived from SEM were strongly correlated with the partial correlation results (*p* < 0.001, **Supplementary Figure 10**). Overall, SMD-induced phenological effects dominated in 35.6% of the TP, while soil moisture effects dominated in 45.5%. Across vegetation types, the soil moisture pathway prevailed in alpine meadow (46.2%) and alpine steppe (52.4%), whereas the SMD-induced phenological pathway was dominant in forests (48.4%) and alpine sparse vegetation (41%).

In summer, snow effects were mediated by six main pathways: three carry-over effects of snow-induced spring growth (**Figures 4a–c**), two phenological pathways (**Figures 4d, e**), and one soil moisture pathway (**Figure 4f**). The carry-over effects of snow-induced spring vegetation growth (Snow affects summer vegetation growth indirectly by first shaping spring growth) dominate 49.6% of the TP (**Figure 4g**). SMD-induced phenological effects play a significant role in 24.6% of the region (**Figure 4d**), whereas snowmelt-driven soil moisture effects govern 12.4% (**Figure 4e**), particularly along the western edge and in parts of the eastern TP. Notably, the phenological effects on vegetation growth in summer is contrary to that in spring, with delayed snowmelt enhancing vegetation growth in summer in the western Himalayas, central alpine steppe, and eastern forest (**Figure 4d**).

Across vegetation types, the carry-over of the soil moisture pathway (i.e., the soil moisture pathway affects summer vegetation growth indirectly by first shaping spring growth) is dominant in alpine steppe (35.4%) and alpine meadow (28.3%), whereas snow-induced phenological effects—arising from both direct and carry-over pathways—prevail in forests and alpine sparse vegetation (**Figure 4h**). Analyses based on SIF show that snow effects mediated by soil moisture are more prominent in both spring and summer than those detected using NDVI (**Supplementary Figures 13**, **S14**), highlighting the stronger role of soil moisture pathways in regulating vegetation productivity across all vegetation types. Nonetheless, the spatial distribution of individual snow effects derived from SIF is broadly consistent with that derived from NDVI.

### 3.3 Transition of dominant pathways

**Figure 5:**
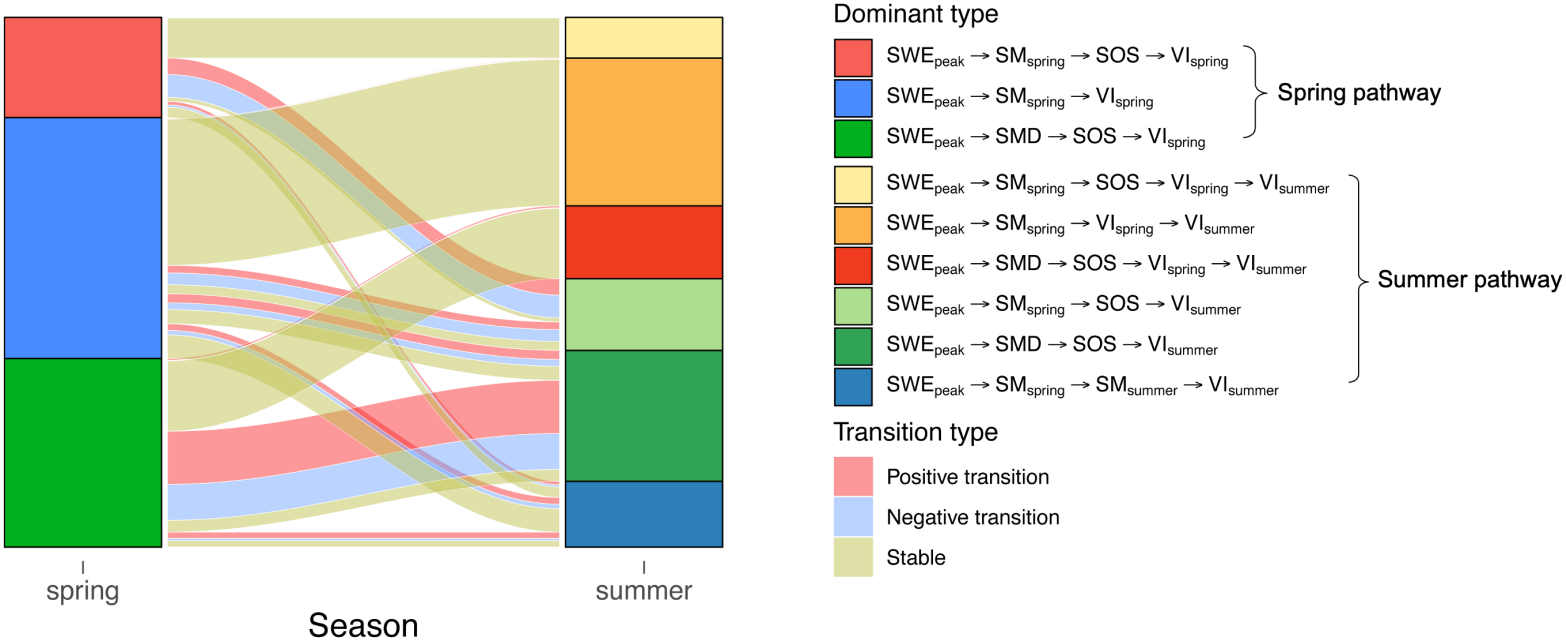
Transition of dominant snow effect types from spring (left column) to summer (right column). The colors of the alluvium indicate transition types (defined by effect values of the dominant snow effect in spring and summer; see **Supplementary Figure 9**). Definitions of transition types are provided in Methods. SOS denotes the start of the growing season, VI denotes NDVI, SMD indicates the snowmelt date, and SM represents soil moisture.

We further quantified the seasonal transitions in dominant pathways. Nearly half of the dominant snow effects (**Supplementary Figure 9**) in summer originated from the carry-over of snow-induced spring vegetation growth (**Figure 5**). Among regions dominated by snow-mediated soil-moisture effects in spring, 61.4% shifted to carry-over effects in summer, while 12.4% transitioned to phenological effects. For regions initially governed by SMD-induced phenological effects in spring, 38.6% continued as phenological carry-over effects (i.e., the SMD-induced phenological pathway affects summer vegetation growth indirectly by first shaping spring growth) in summer, 53.5% shifted to direct phenological effects (i.e., the SMD-induced phenological pathway exerts a direct influence on summer vegetation growth), and 7.9% transitioned to soil moisture effects. Despite the overall direction of snow effects remaining unchanged from spring to summer in 63.4% of the region, the underlying processes undergo substantial shifts. Among these stable regions, 76.4% were characterized by spring snow effects that persisted into summer as carry-over pathways, and within this subset, 57% represented the carry-over of the soil moisture pathway. Positive transitions in snow effects accounted for 20% of the TP, mainly reflecting the reversal of phenological effects—among which transitions driven by snowmelt date (SMD-induced) contributed 50.2%, and those driven by soil moisture (SM-induced) contributed 15.6%. Conversely, negative transitions covered 16.6% of the TP, also largely dominated by reversed phenological pathways, with SMD-induced and SM-induced transitions accounting for 40.9% and 25.8%, respectively.

### 3.4 Comparison of snow effects between observation and model simulations

**Figure 6:**
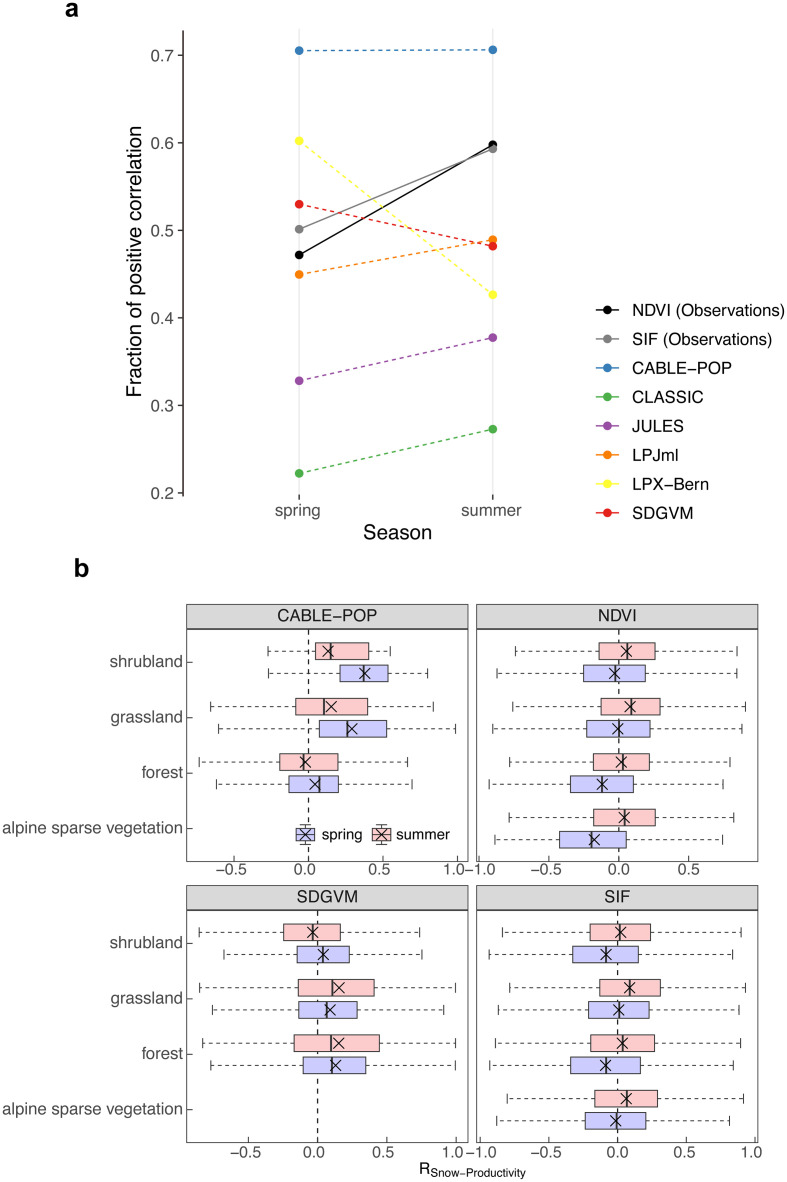
Comparison of satellite-derived observations and TRENDY model simulations of seasonal transitions in snow effects on vegetation growth. **(a)** Percentage of pixels showing positive partial correlations between winter snow (quantified by snow depth_peak_ or SWE_peak_) and seasonal vegetation productivity (quantified by GPP in TREDNY model) during the period of 2000–2017. **(b)** Partial correlation between winter snow and seasonal vegetation productivity (R_snow-productivity_) across vegetation types.

Comparison with partial correlations derived from the TRENDY ensemble (**Supplementary Figure 16**) reveals substantial discrepancies between model simulations and remote-sensing observations. Considerable inter-model spread is evident among TRENDY simulations. Specifically, CABLE-POP tends to overestimate the positive effects of snow on vegetation growth in both spring and summer, whereas CLASSIC and JULES underestimate them. The pronounced seasonal transition in snow effects identified from observations is only weakly reproduced by a subset of models (i.e., LPJml, JULES, and CLASSIC; **Figure 6a**), and the magnitude of this transition is systematically underestimated. In contrast, LPX-Bern and SDGVM simulate an opposite seasonal pattern. Satellite-based observations from SIF and NDVI further indicate marked seasonal differences in snow effects across vegetation types. However, in models that explicitly represent snow processes for individual plant functional types (PFTs) within each tile, these vegetation-specific seasonal differences are largely absent (**Figure 6b**).

## 4 Discussion

### 4.1 Pervasive snow-induced carry-over effects between spring and summer

We identify the core pathways through which winter snow influences vegetation growth across seasons. In spring, snow affects vegetation growth primarily through two pathways: the phenological pathways (driven by SMD) and soil moisture pathway (indirectly driven by snow mediated soil moisture). Specifically, winter snow accumulation enhances spring vegetation growth in 53.4% of the TP via the soil moisture pathway, with prominent effects in Qinghai-lake basin (**Figure 3b**), where SWE_peak_ significantly increased during 2000-2017 (**Supplementary Figure 5a**). In contrast, winter snow exhibits a negative effect on vegetation growth in 55.6% of the TP via the SMD-induced phenological pathway, an effect especially pronounced in forests across the eastern TP (**Figure 2a** and **3c**), where SMD significantly advanced phenology (**Supplementary Figure 5b**). Meanwhile, the soil-moisture–induced phenological pathway (SWE_peak_ altered soil moisture, which in turn affected spring phenology and hence NDVI) shows a positive relationship with NDVI in 54.2% of the TP (**Figure 3a**). These results align with previous findings that grasslands exhibit the strongest positive response to soil moisture, whereas forests show the most pronounced negative response to snow phenology (Wang et al., 2024). The spring increase in growth and carbon uptake of coniferous forests is particularly sensitive to the timing of snowmelt (Pulliainen et al., 2017; Parazoo et al., 2018), which corroborates our finding of a significant negative snow–vegetation relationship in the forests at eastern TP during spring. The soil moisture pathway is most prominent in the northeastern TP, where water limitation strongly constrains ecosystem productivity (Xu et al., 2021; Meng et al., 2024). In these dry regions, snow provides significant water supply that facilitates vegetation growth (Wu et al., 2019).

In summer, snow influences vegetation growth mainly through the carry-over of its phenological and soil moisture effects in spring, which dominated 49.6% of TP. Each spring pathway extends into summer as a carry-over pathway, generally exerting a homodromous influence on vegetation growth, thereby contributing to the regions where the direction of dominant snow effects remains stable. The soil moisture effect of snow shows a pervasive positive impact on vegetation growth and often persists over relatively long durations, especially at colder regions characterized with short growing season and high quantity of snowpack meltwater (Wang et al., 2018; Liu et al., 2023a; Grippa et al., 2005). This supports our finding that soil moisture effects induced by snow are more likely to be carried over into the next season. Moreover, previous studies have shown that insufficient snow in the pre-growing season can suppress tree growth or enhance tree growth sensitivity to water stress during the growing season (D’Orangeville et al., 2018; Wu et al., 2022), likely due to the weakening of the carry-over of the soil moisture pathway. This pathway is further supported by plot-scale studies in Arctic tundra, where higher snow accumulation has been linked to greater peak NDVI during the growing season (Moriana-Armendariz et al., 2022; Pirk et al., 2023). Our SIF-based results reveal a much stronger dominance of soil moisture effect despite of seasons than NDVI-based results, suggesting that the snow impact on vegetation productivity is underestimated when vegetation greenness is used as a proxy. This limitation is common in large-scale studies of snow effects, probably due to the saturation feature of NDVI. Incorporating SIF or GPP may therefore provide new opportunities for advancing snow–vegetation productivity research.

### 4.2 Seasonal transition in snow effects

This study reveals substantial transitions in the snow–vegetation growth relationship from spring to summer. Two major transition modes are identified across the TP: positive transitions (20% of the area) and negative transitions (16.6%). For positive transitions, about half are driven by a reversed SMD-induced phenological effect, characterized with shifting from negative effects on vegetation growth in spring to positive in summer. In spring, advances in SMD can result in an earlier spring phenology and thus enhance vegetation growth, consistent with large-scale observations across regions north of 30°N in the Northern Hemisphere (Wang et al., 2018). However, this phenological effect reverses in summer, where earlier SMD leads to decrease in vegetation growth. Study using satellite data also shown warm spring had negative lagged effect on NDVI in summer in Siberia region (Grippa et al., 2005; Buermann et al., 2018). The underlying mechanisms behind this divergent seasonal pattern likely involve more than one process. On one hand, the reversal likely reflects soil moisture depletion in summer, exacerbated by earlier spring phenology, a phenomenon widely observed in snow-dominated northern latitudes (Lian et al., 2020; Zhang et al., 2021). On the other hand, cold ecosystems may exhibit adaptive responses to climatic constraints, as reflected in NDVI trajectories (Park et al., 2019). For instance, Arctic plants often show phenological timing determined by snowmelt patterns, whereas the duration of phenological phases remains fixed (Semenchuk et al., 2016). Consequently, early snowmelt advances the entire NDVI trajectory, thereby generating the apparent seasonal divergence in snow effects (**Supplementary Figures 11d**, **12c**). However, when we examine these effects over the entire growing season, it appears that the negative effects in spring tend to outweigh and persist throughout the entire growing season. For negative transitions, nearly half are caused by a reversed SMD-induced phenological pathway, shifting from positive in spring to negative in summer. These are mainly observed in alpine grasslands (**Supplementary Figure 15**). Another one-quarter of the negative transitions result from a reversed soil-moisture–induced phenological pathway. In arid regions such as western China, increased soil moisture can advance phenology and stimulate early-season growth (Chen et al., 2019; Wu and Liu, 2013), making these ecosystems prone to soil-moisture–induced reversals of phenological effect. In snow-dominated ecosystem, productivity is closely tied to snowmelt timing (Parazoo et al., 2018; Park et al., 2019; Pulliainen et al., 2017). These seasonal transitions in snow effects may complicate the overall influences of snow on vegetation growth and ecosystem productivity. As previous study have highlight on water supplement in snow (Peng et al., 2010; Hu et al., 2013; D’Orangeville et al., 2018), while the negative phenological effect is more pronounced sometimes. We revealed snow effects extend to the growing season, with significant seasonal signals more likely to persist, such that regions with spring suppression or summer enhancement tend to exhibit consistent relationships at the growing season scale. This suggests that the overall effects of snow depend on local environmental conditions, specifically on which pathways vegetation is most sensitive to.

Notably, TRENDY simulations show a limited ability to reproduce the observed seasonal transitions in snow effects on vegetation growth on the TP, with substantial inter-model uncertainty evident. Some models partially capture the observed transition, whereas others exhibit contrasting or even opposite seasonal patterns. These discrepancies may partly arise from an underestimation of snow-induced phenological effects in certain models (Wang et al., 2018, Wang et al., 2024), or from an insufficient representation of the carry-over of the soil moisture pathway into summer (Hu et al., 2013; D’Orangeville et al., 2018). Moreover, large discrepancies in the spatial patterns of snow effects persist between TRENDY simulations and observations (**Supplementary Figure 16**), indicating that the representation of ecosystem-specific snow effects remains inadequate.

This study is based on remote-sensing observations and uses partial correlation and piecewise SEM to generate patterns of snow effects. The results from the two methods corroborate each other in spring (**Supplementary Figure 10**), but are mediocre in summer, indicating that the positive transition observed by partial correlation can only be partially explained by piecewise SEM. The effects of snow on subsequent vegetation growth are complex, especially with regard to its insulation effects on soil temperature and nutrient-related processes, which were not considered in this study. However, the lack of soil nutrient data hinders our ability to detect these effects at large scale. In the model comparison, we used GPP and snow depth in most models due to the unavailability of NDVI or snow water equivalent. This could introduce some uncertainties into the comparisons. Despite this, SIF, which is closely related to GPP, shows results consistent with those derived from NDVI.

Nevertheless, this study provides comprehensive understanding of how winter snow influences alpine vegetation growth through multiple, seasonally dependent pathways across the Tibetan Plateau. By disentangling the effects of peak snow water equivalent (SWE_peak_) and snowmelt timing, we reveal transitions in snow–vegetation relationships from spring to summer, reflecting shifts among phenological, soil moisture, and carry-over processes—dynamics that are currently not well captured in dynamic global vegetation models. These findings highlight that snow effects are not uniform but vary with seasons, vegetation types, and hydrothermal conditions, leading to contrasting ecological outcomes. As snow regimes continue to alter under a warming climate (Musselman et al., 2021; Wieder et al., 2022), these results underscore the need to account for the seasonal dynamics of snow effects in phenological and snow-related modelling research.

## Data Availability

The original contributions presented in the study are included in the article/**Supplementary Material**. Further inquiries can be directed to the corresponding author.
