# Supplementary material for: Seasonal transition in snow effects on vegetation growth across the Tibetan Plateau
Source: Front Plant Sci. 2026 Jul 13;17:1855382. doi: 10.3389/fpls.2026.1855382 (PMC13402452; doi:10.3389/fpls.2026.1855382)
Supplement: Supplementary file 1 [file DataSheet1.docx]

**Seasonal transition in snow effects on vegetation growth across the Tibetan Plateau**

Zekai Meng^1,2^, Xiuchen Wu^3,*^, Yongmei Huang^1,2^, Renjie Guo^1,2^, Xiaona Wang^1,2^, Wenqi Song^1,2^, Zifan Zhang^1,2^

State Key Laboratory of Earth Surface Processes and Disaster Risk Reduction, Beijing Normal University, Faculty of Geographical Science, Beijing, 100875, China.

2 School of Natural Resources, Beijing Normal University, Faculty of Geographical Science, Beijing, 100875, China.

3 Department of Health and Environmental Sciences, School of Science, Xi’an Jiaotong-Liverpool University, Suzhou, 215123, China

* To whom the correspondence should be addressed, Xiuchen Wu, Department of Health and Environmental Sciences, School of Science, Xi’an Jiaotong-Liverpool University, Suzhou, 215123, China. E-mail: [xiuchen.wu@xjtlu.edu.cn](mailto:xiuchen.wu@xjtlu.edu.cn); Tel: 0086-512-8816 1330; Fax: 0086-512-8816 1330

**This file includes Figures S1-16.**


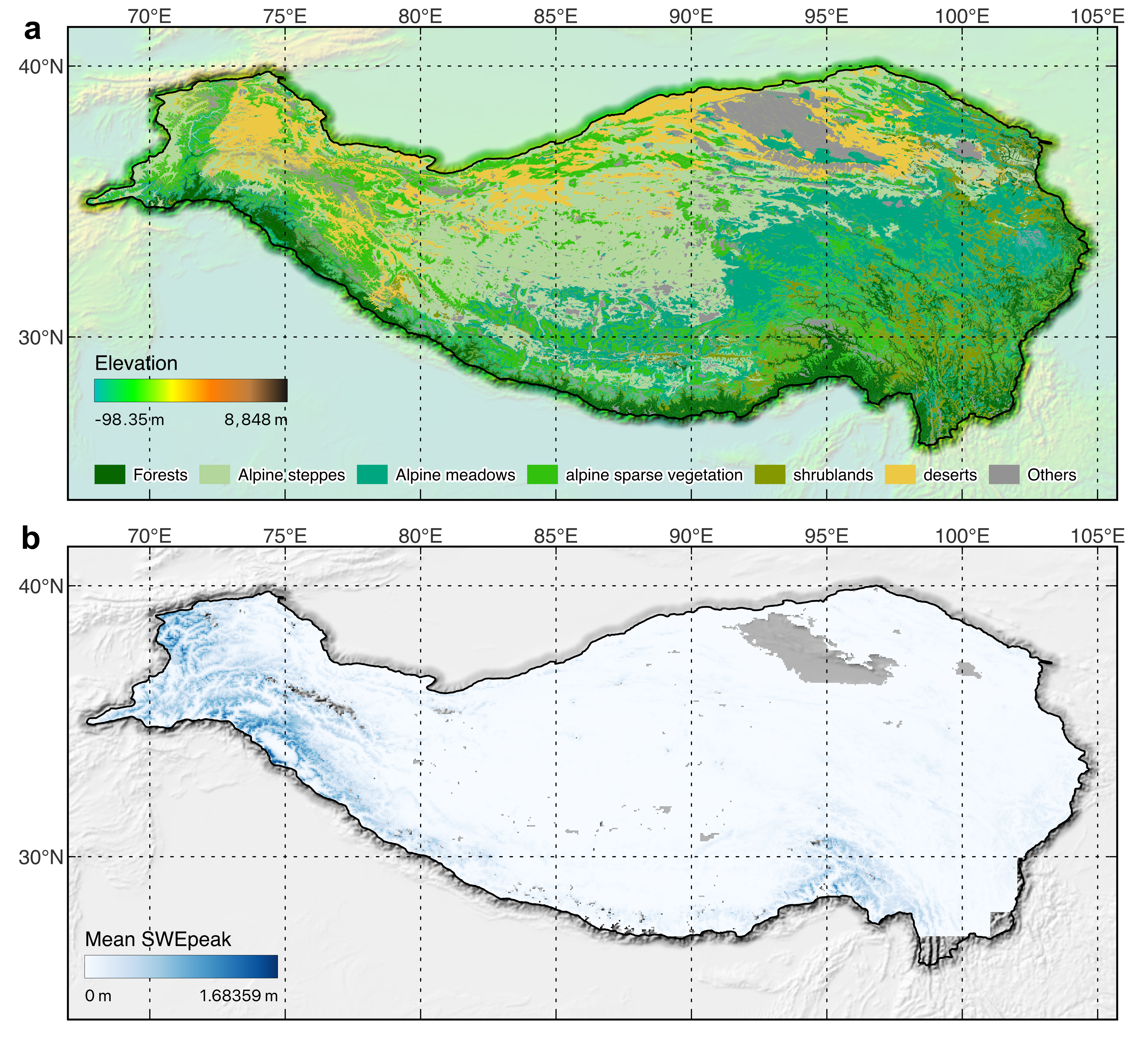


**Figure S1. The spatial distribution of study region.** The spatial distribution of different vegetation types (a) and snow regime (b) were displayed. This study focused on four major vegetation types, including forests, shrublands, alpine meadows and alpine steppes.

**Figure S2. NDVI fitting with the quality assurance (QA) layer.** Biweekly NDVI versus time with colors indicating QA (a), and comparison before and after NDVI smoothing (b). The example pixel is located in a forested area of the Tibetan Plateau.

**Figure S3. Distribution of each phenological phase across Tibetan Plateau.** used in defining seasons. Spring was defined as the period of [UD_m_, SOS_m_], and summer as (SOS_m_, EOS_m_) (definition see methods).


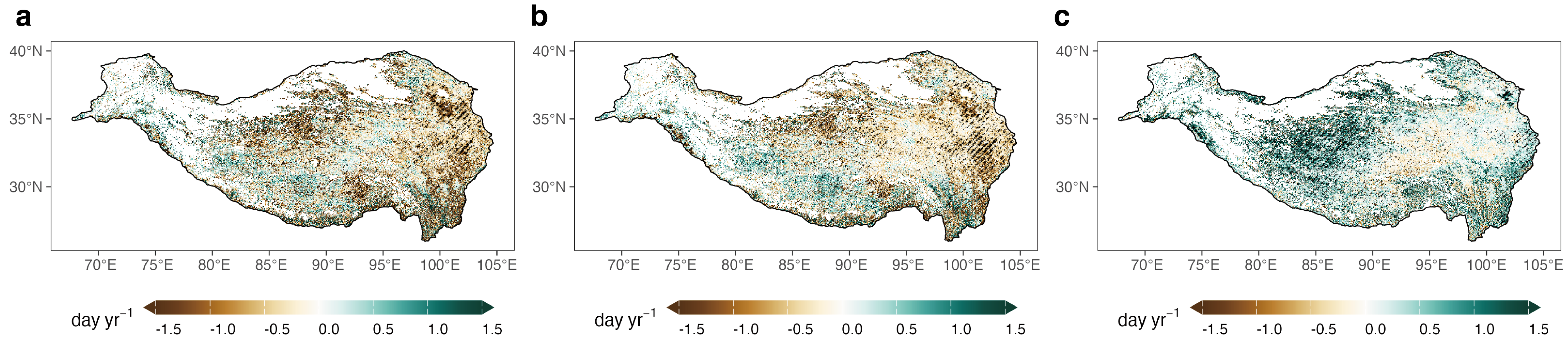


**Figure S4. Trend of phenology during 2000-2017 on the TP.** Trend of UD (a), SOS (b), EOS (c) derived from mean of 4 smoothing methods (see methods). Maps were generated using Sens slope and Mann-Kendall test. Areas with line patterns indicate significant trend (Mann-Kendall test p-value < 0.05)


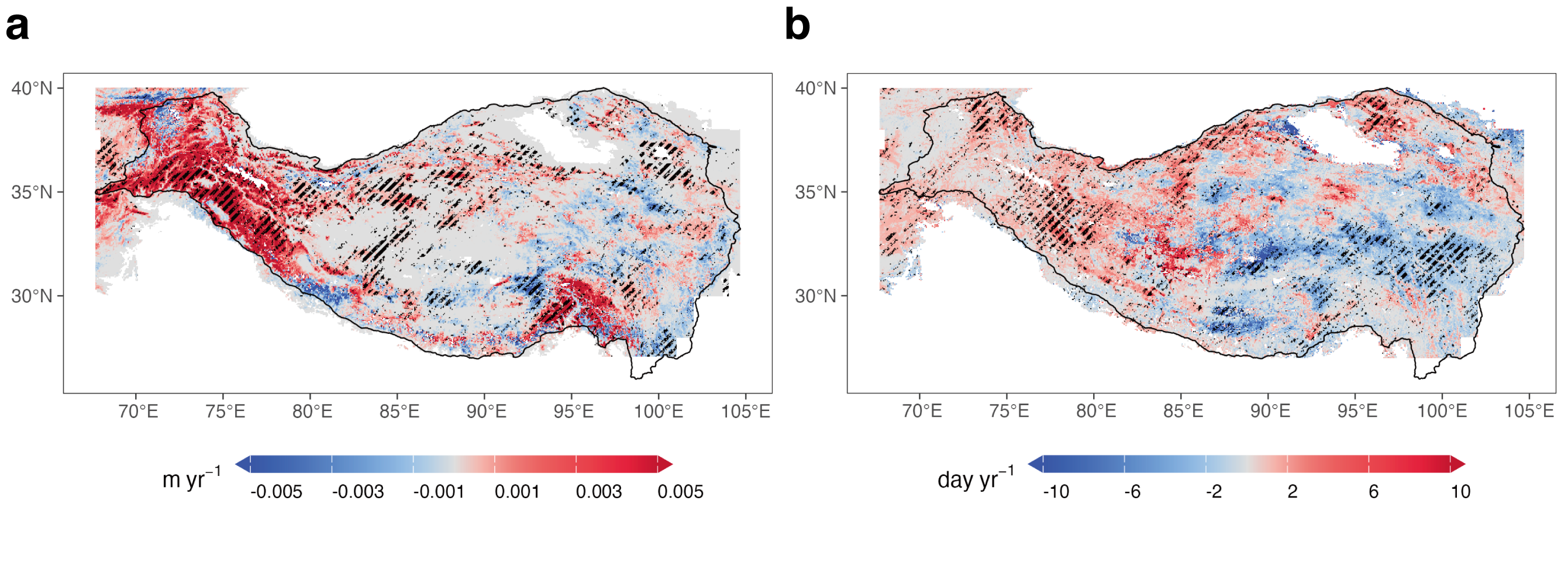


**Figure S5. Trend of snow parameters during 2000-2017 on the TP.** Trend of SWE_peak_ (a), snow melt date (b). Maps were generated using Sens slope and Mann-Kendall test. Areas with line patterns indicate significant trend (Mann-Kendall test p-value < 0.05)


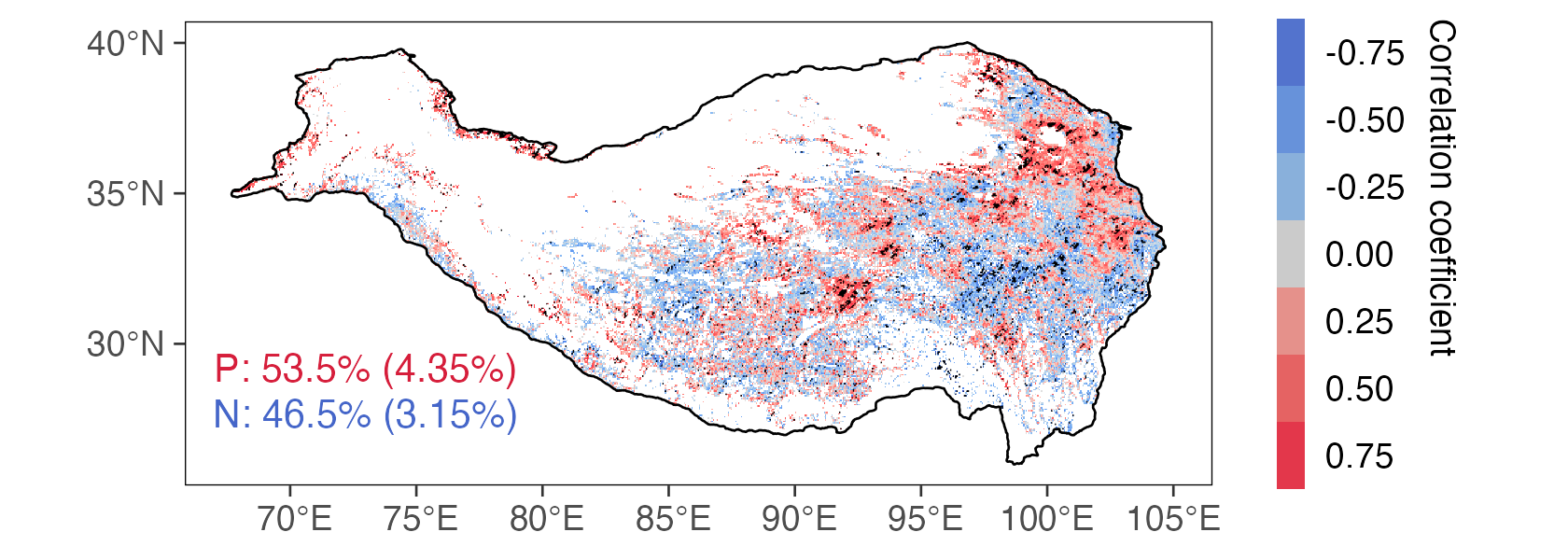


**Figure S6. Effects of snow on growing season NDVI**. Spatial patterns of the partial correlation coefficient between SWE_peak_ and NDVI (R_SWE-NDVI_) during growing season(a). The partial correlation controls for concurrent spring temperature, spring VPD, spring precipitation, and spring shortwave radiation. P, positive correlation; N, negative correlation. In each panel, overall percentages of positive/negative correlations are given, with significant proportions in parentheses. Areas with line patterns indicate significant R_SWE–NDVI_ relationships (P < 0.05).


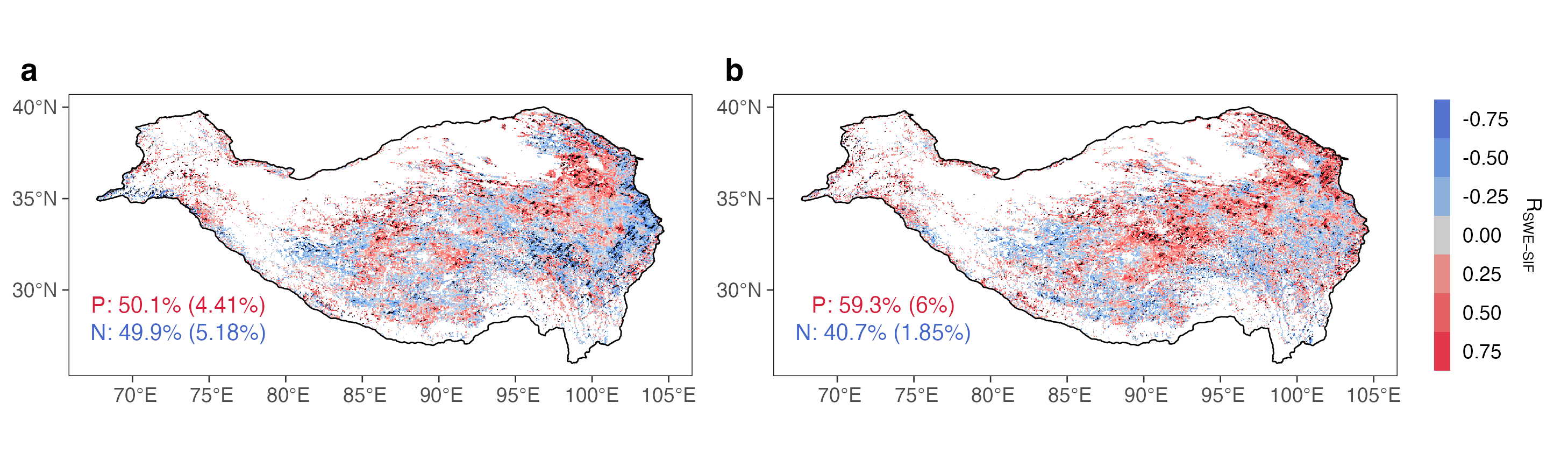


**Figure S7. Spatial patterns of the partial correlation coefficient between SWE_peak_ and seasonal mean SIF in spring (a) and summer (b).** The spring analysis controls for spring temperature, VPD, precipitation, and shortwave radiation, while the summer analysis additionally accounts for precipitation in the spring. P, positive effect; N, negative effect. In each panel, the overall percentages of positive and negative correlations are given, with significant proportions in parentheses. Areas with line patterns indicate significant R_SWE–SIF_ relationships (P < 0.05).


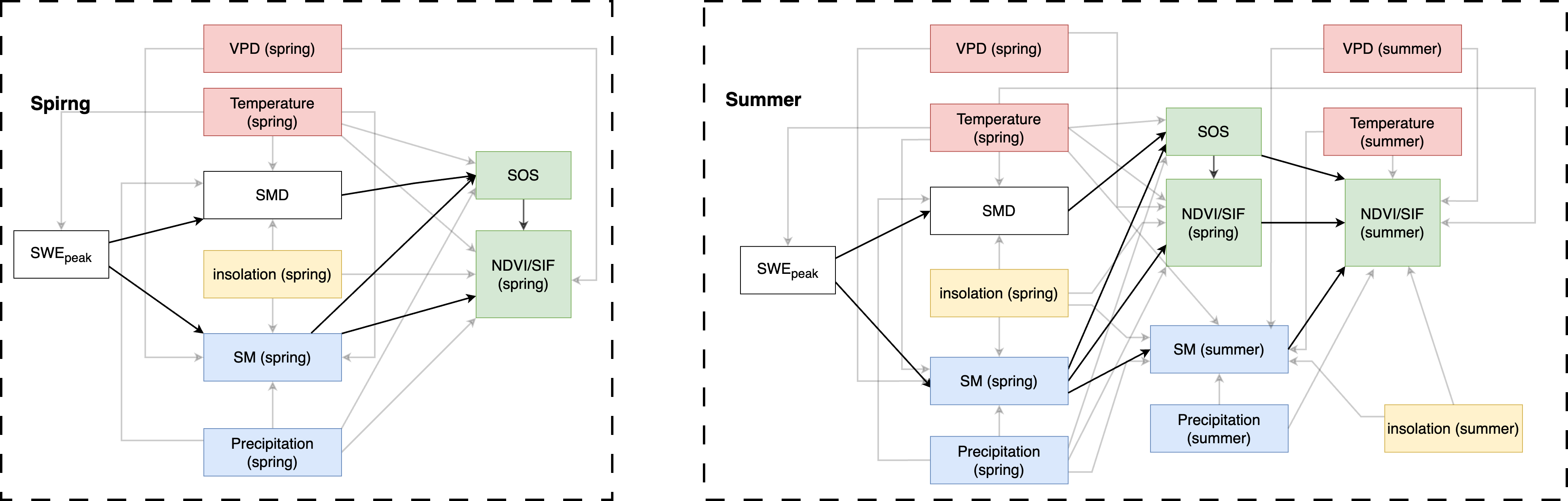


**Figure S8. Final structural equation models (SEM) for spring and summer.** The diagrams illustrate the mechanisms through which peak snow water equivalent (SWE_peak_) influences seasonal productivity. For SWE_peak_, snowmelt date (SMD), and start of the growing season (SOS), same values were provided for both seasons. For temperature, shortwave radiation (insolation), soil moisture (SM), and NDVI, seasonal means were used, while precipitation was represented by seasonal totals. Black solid lines indicate direct pathways related to snow effects on vegetation growth. Other pathways are not directly linked to snow but are included to disentangle the overall snow influence. Final SEMs were applied to each pixel to disentangle snow effects (see in Methods).

The simplified code below demonstrates all the pathway in the spring and summer model explicitly.

Rcode:

Summer_model <- psem(

lm(VI_summer ~ SM_summer + prec_summer + temp_summer + srad_summer + VI_spring + SOS + temp_spring +VPD_summer, d),

lm(VI_spring ~ temp_spring + SOS + srad_spring + SM_spring + prec_spring + VPD_spring, d),

lm(SM_summer ~ SM_spring + prec_summer + srad_summer +temp_spring + temp_summer + srad_spring + prec_spring + VPD_summer, d),

lm(SM_spring ~ prec_spring + SWE_max + srad_spring + temp_spring + VPD_spring, d),

lm(SOS ~ SMD + temp_spring + SM_spring + prec_spring, d),

lm(SMD ~ SWE_max + prec_spring+ srad_spring + temp_spring, d),

lm(SWE_max ~ temp_spring, d)

)

Spring_model <- psem(

lm(VI_spring ~ SOS + SM_spring + prec_spring + temp_spring + srad_spring + VPD_spring, d),

lm(SOS~ SMD+temp_spring + SM_spring + prec_spring, d),

lm(SWE_max ~ temp_spring, d),

lm(SMD ~ SWE_max + temp_spring+ prec_spring + srad_spring, d), #

lm(SM_spring ~ SWE_max + prec_spring + temp_spring + srad_spring + VPD_spring, d)

)


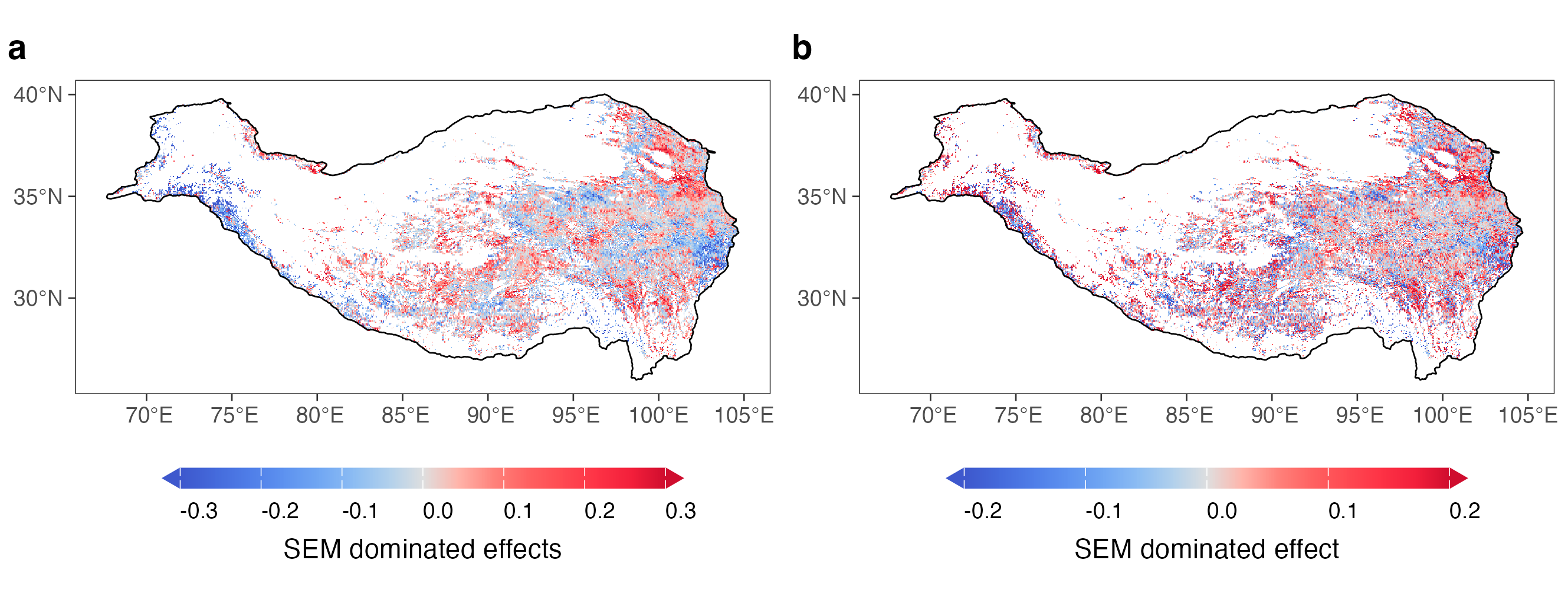


**Figure S9. SEM-derived dominant snow effects in spring (a) and summer (b),** calculated as the effect values (derived by multiplying standardized coefficients of direct pathways that formed an indirect pathway in SEM) of the dominant disentangled snow effect type in each season.


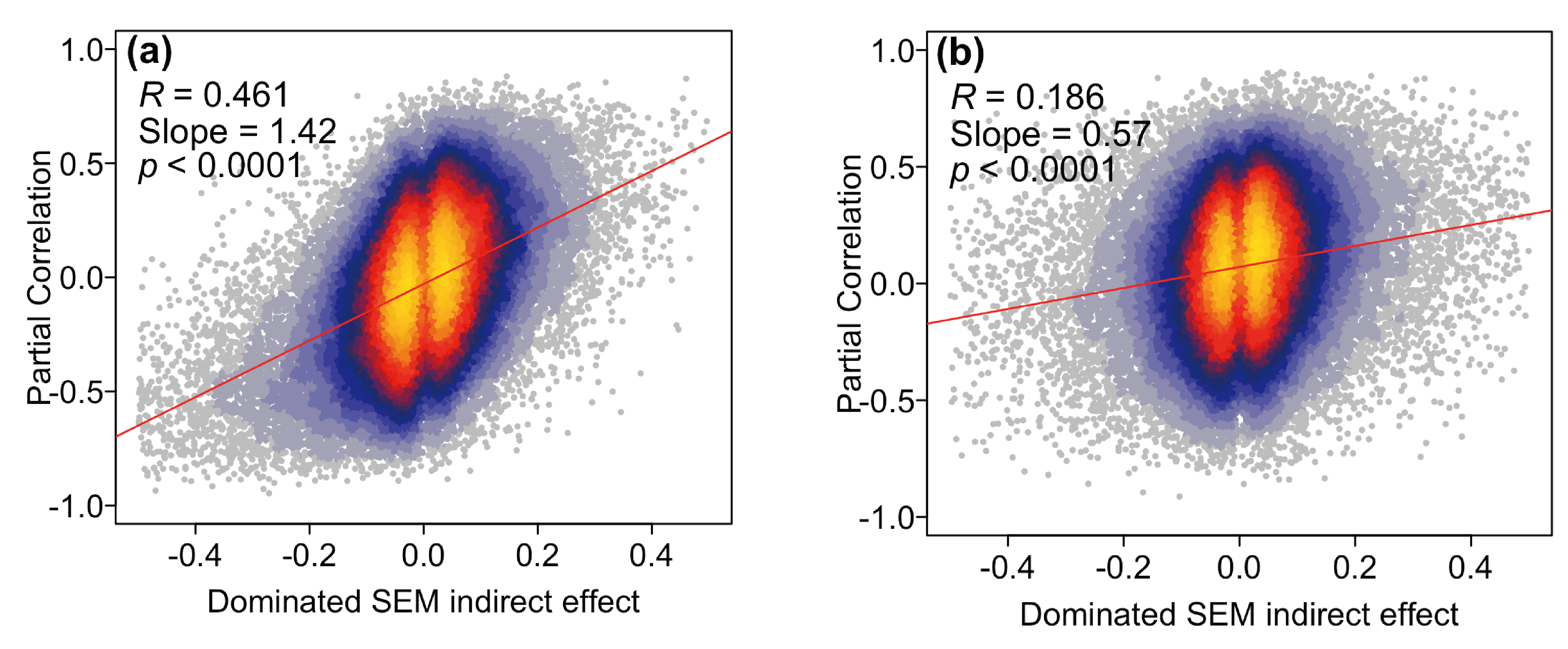


**Figure S10. Comparison between snow effects quantified by partial correlation and by SEMs (Figure 2 vs. Figure S9) in spring (a) and summer (b).**


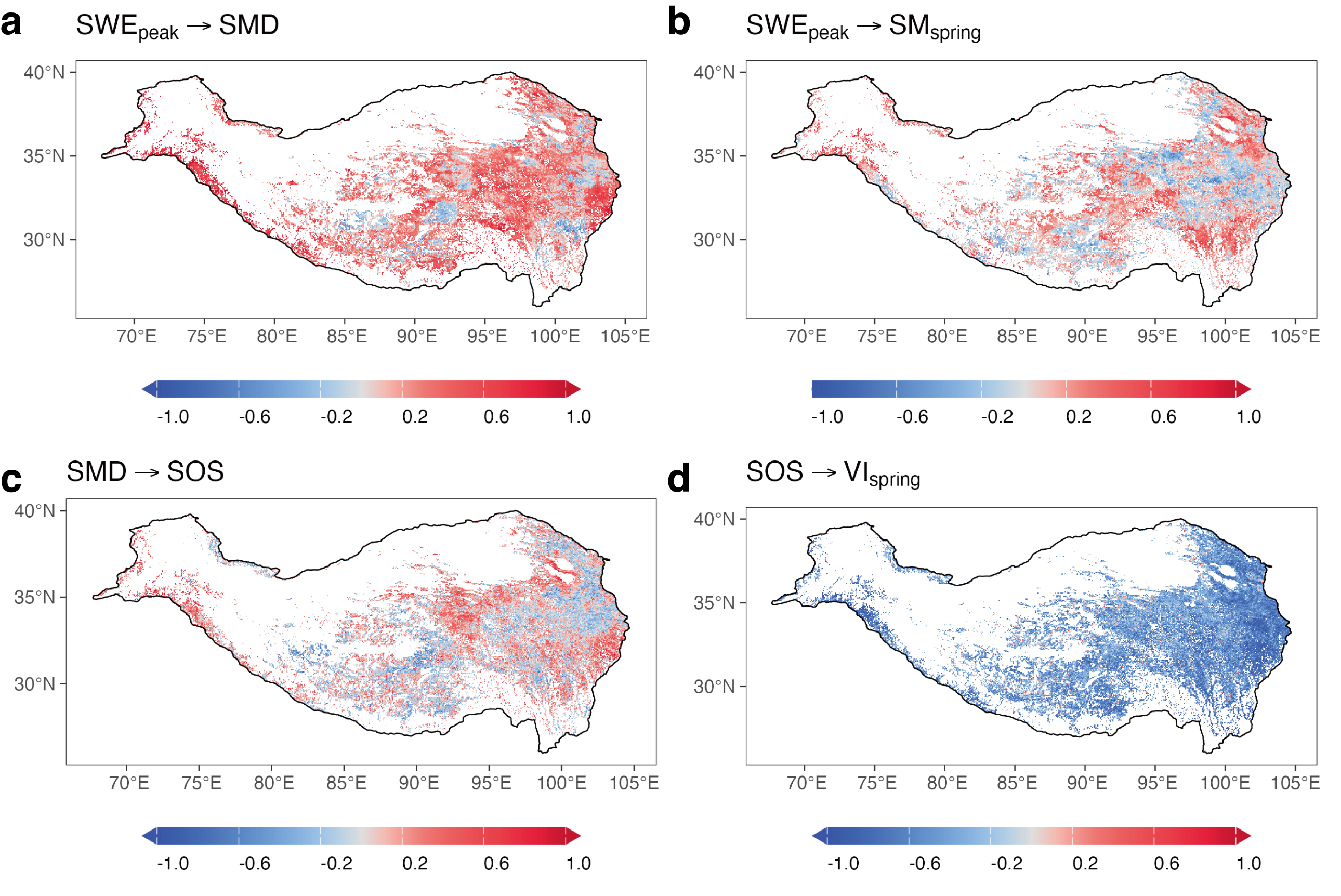


**Figure S11. Core direct pathways coefficient that constitutes indirect pathways in spring.**


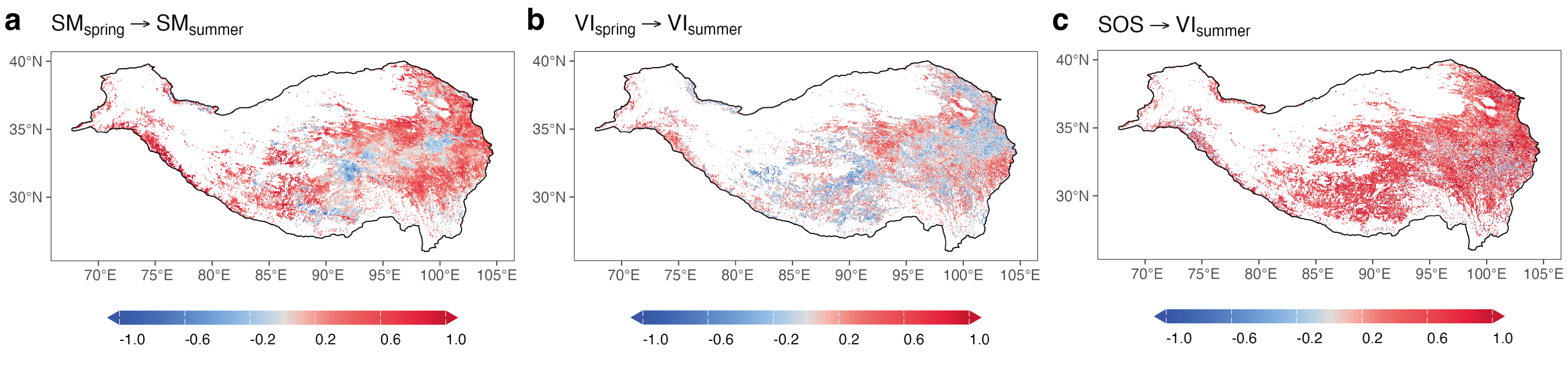


**Figure S12. Core direct pathways coefficient that constitutes indirect pathways in summer**


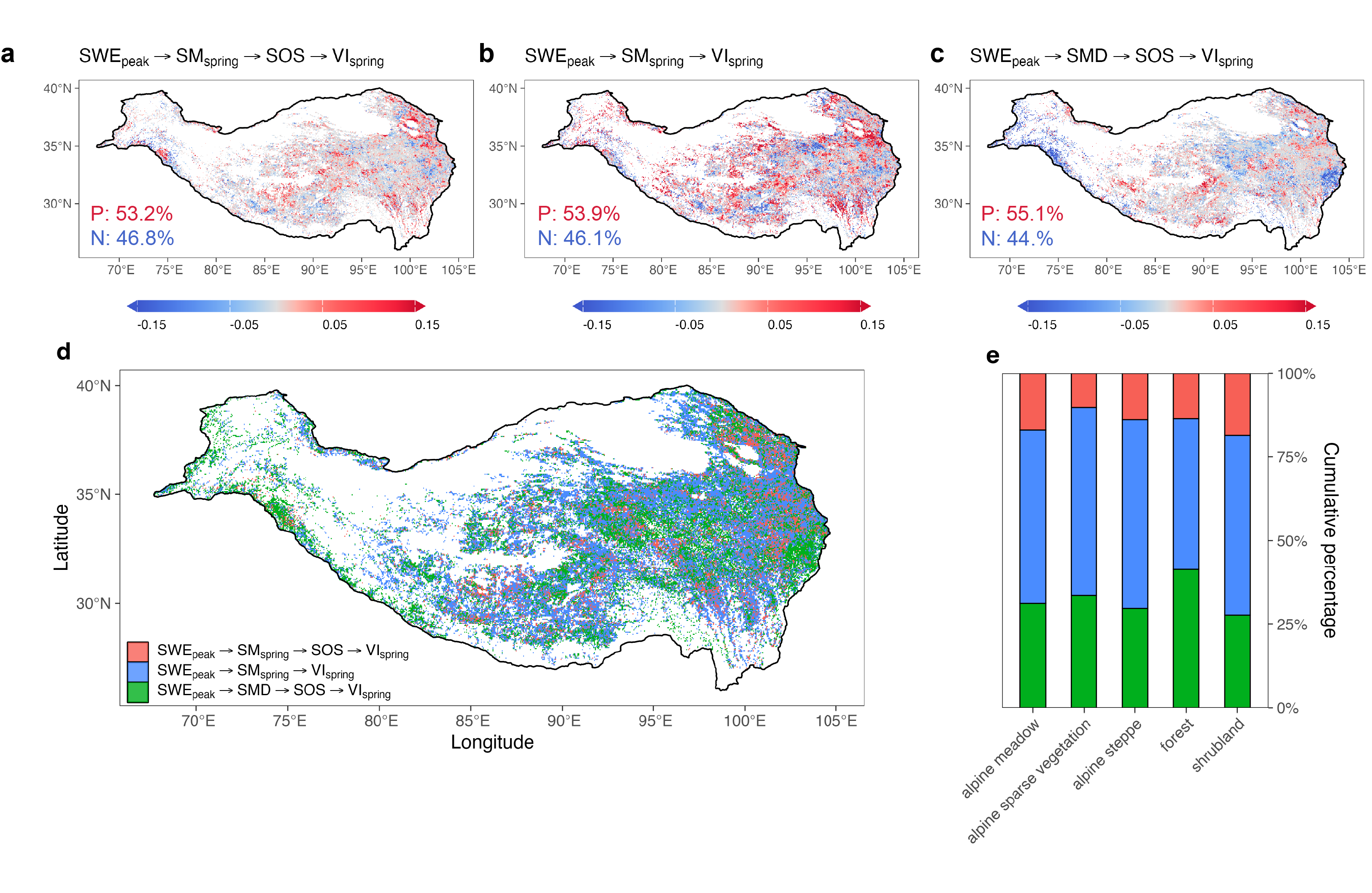


**Figure S13. Pathways through which snowmelt influences SIF in spring.** Spatial patterns of indirect effects of SWE_peak_ on NDVI via different pathways (a–c), spatial pattern of dominant snow effect types (d), and their distribution across vegetation types (e). SOS denotes the start of the growing season, VI denotes SIF, SMD indicates the snowmelt date, and SM represents soil moisture.


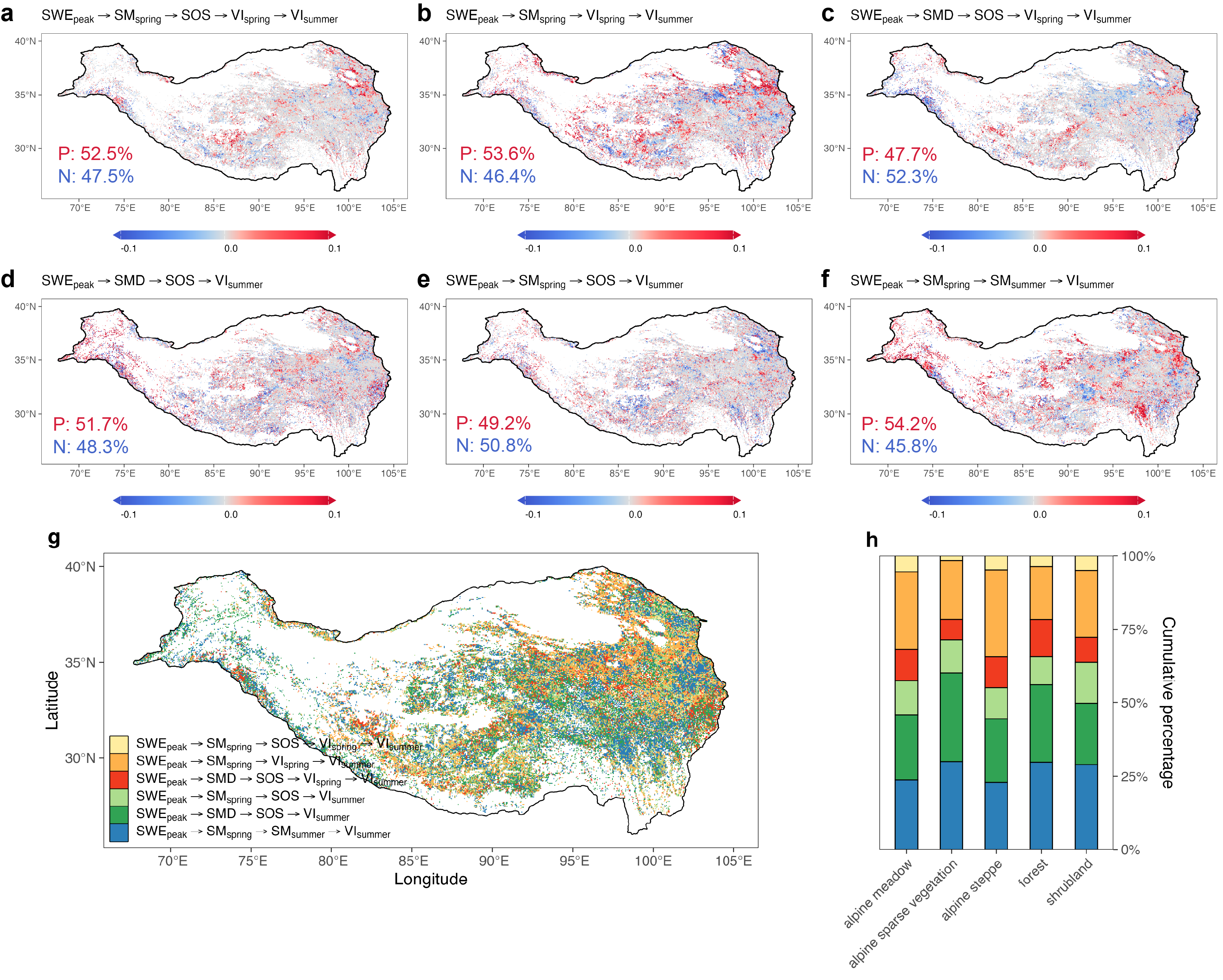


**Figure S14. Pathways through which snowmelt influences SIF in summer.** Spatial patterns of indirect effects of SWE_peak_ on NDVI via different pathways (a–f), spatial pattern of dominant snow effect types (g; warm colors indicate carry-over effects of spring snow), and their distribution across vegetation types (h). SOS denotes the start of the growing season, VI denotes SIF, SMD indicates the snowmelt date, and SM represents soil moisture.


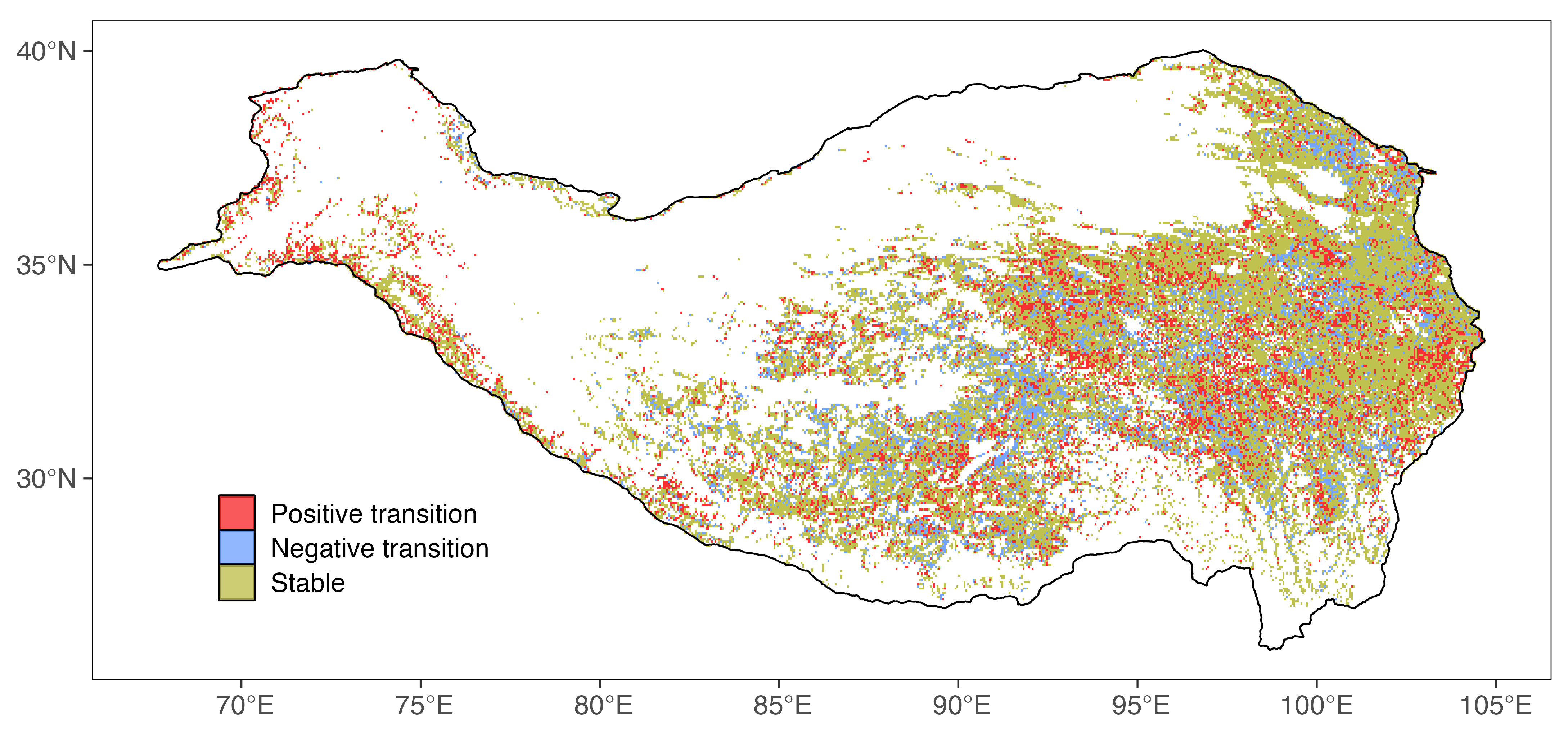


**Figure S15. Transitions in the SEM-derived dominant snow effects.** A positive transition indicates a shift in the coefficient from negative in spring to positive in summer, whereas a negative transition represents the opposite pattern. Non transition indicates that the sign of the coefficient remains unchanged between seasons.





**Figure S16. Spatial patterns of partial correlation coefficients between snow (quantified by snow depth or snow water equivalent, depending on data availability) and gross primary production (GPP) in spring (left column) and summer (right column) derived from different TRENDY model simulations.** The spring analysis controls for concurrent temperature, precipitation, and downward shortwave radiation, while the summer analysis additionally accounts for precipitation in the preceding season.

# DATA AVAILABILITY STATEMENT

The data and code that supports the findings of this study are available from Zenodo at <https://zenodo.org/records/18387531>. Daily snow water equivalent (SWE) data from this study is available at <https://nsidc.org/data/hma_sr_d/versions/1>. The MOD13A2 NDVI product is available at <https://lpdaac.usgs.gov/products/mod13a2v006/>. Contiguous SIF (CSIF) dataset is available at <https://data.tpdc.ac.cn/zh-hans/data/d7cccf31-9bb5-4356-88a7-38c5458f052b/>. TerraClimate data are available at <https://www.climatologylab.org/terraclimate.html>. Soil moisture data from FLDAS v4 is available at <https://disc.gsfc.nasa.gov/datasets/FLDAS_NOAH01_C_GL_M_001/summary>. Vegetation type data is available at <https://data.tpdc.ac.cn/zh-hans/data/0b32907c-9f64-407b-8099-849db1900005>. TRENDY v12 data are available at <https://mdosullivan.github.io/GCB/>.
